# Severe symptoms and very low quality-of-life among outpatients newly diagnosed with advanced cancer: data from a multicenter cohort study

**DOI:** 10.1007/s00520-020-05388-y

**Published:** 2020-03-17

**Authors:** Waldemar Siemens, Stefan S. Schönsteiner, Claudia Lorena Orellana-Rios, Ulrike Schaekel, Jens Kessler, Corinna Eschbach, Marén Viehrig, Regine Mayer-Steinacker, Gerhild Becker, Jan Gaertner

**Affiliations:** 1 Clinic for Palliative Care, Medical Center, Faculty of Medicine, University of Freiburg, Robert-Koch-Str 3, 79106 Freiburg, Germany; 2 Department of Internal Medicine III, University Hospital Ulm, Ulm, Germany; 3 Internal Medicine V, Hematology/Oncology/Rheumatology, Heidelberg University Hospital, Heidelberg, Germany; 4 Department of Anesthesiology, Center of Pain Therapy and Palliative Care Medicine, University Hospital Heidelberg, Heidelberg, Germany; 5 Department of Thoracic Oncology, Member of the German Centre for Lung Research (DZL), University Hospital Heidelberg and Translational Lung Research Center Heidelberg (TLRC-H), Heidelberg, Germany; 6 Department of Radiooncology, Palliative Care Unit, University Hospital of Tübingen, Tuebingen, Germany; 7 Center for Palliative Care Hildegard, Basel, Switzerland

**Keywords:** Palliative care, Early palliative care, Quality of life, Symptom assessment, Neoplasms

## Abstract

**Purpose:**

The aim of this study was to identify symptoms of *severe* intensity or *very low* scores for quality of life (QoL) domains in newly diagnosed outpatients with advanced cancer.

**Methods:**

This multicenter cohort study from a state-wide palliative care network included adult outpatients with advanced cancer diagnosed within the preceding 8 weeks from four comprehensive cancer centers (DRKS00006162, registered on 19 May 2014). We used the Palliative Outcome Scale (POS), Hospital Anxiety and Depression Scale, and European Organization for Research and Treatment of Cancer QoL Questionnaire-C30. For each questionnaire, cut-off scores defined symptoms and QoL domains that were considered “severe” or “very low.”

**Results:**

Of 3155 patients screened, 481/592 (81.3%) were analyzed (mean age 62.4; women *n* = 245, 50.9%). We identified 324/481 (67.4%) patients experiencing at least one severe symptom or a very low QoL domain (median 2; range 0 to 16). *Role functioning* (*n* = 180, 37.4%), *fatigue* (*n* = 162, 33.7%), and *social functioning* (*n* = 126, 26.2%) were most commonly affected. *QoL* was very low in 89 patients (18.5%). Women experienced more anxiety symptoms, fatigue, and had lower POS scores. Patients often mentioned physical symptoms and fears of adverse events resulting from disease-modifying therapies (e.g., chemotherapy) as most relevant problems.

**Conclusions:**

Already within the first 8 weeks after diagnosis, the majority of patients reported at least one *severe* symptom or a *very low* QoL domain. Gender differences were evident. The findings illustrate the value of early routine assessment of patient burden and the development of multi-professional and interdisciplinary palliative care.

**Electronic supplementary material:**

The online version of this article (10.1007/s00520-020-05388-y) contains supplementary material, which is available to authorized users.

## Introduction

Cancer patients experience a large variety of symptoms throughout the course of their disease and it is becoming increasingly recognized that to some extent, symptoms may be relevant already early after diagnosis of advanced cancer for some patients. Kaasa and Loge et al. [1] proposed the use of standardized care pathways and multidisciplinary teams to foster the integration of oncology and palliative care teams already early after diagnosis of advanced cancer to respond timely to patients’ needs with a coordinated approach of specialist teams.

Some studies assessed symptoms and burden of patients with either a large time variation since cancer diagnosis [2] or cancer progression [3]. Others primarily focused on the prevalence or mean intensity of symptoms and quality of life (QoL) in general [4] or at the end of life [3, 5–7]. Knowing symptom intensity and the QoL early after diagnosis of advanced cancer is highly relevant to optimize cancer care and to allocate resources according to patients’ needs [1].

The aims of the study presented here were therefore to identify (i) how many patients with newly diagnosed advanced cancer suffered from symptoms of *severe intensity* and *very low* QoL domains and (ii) which symptoms and QoL domains were the most frequent causes for such suffering [8].

## Methods

### Study design

In this cross-sectional analysis from a multicenter cohort study (EVI project, DRKS00006162, registered on 19 May 2014) [8], we enrolled outpatients from 23 oncology departments from four comprehensive cancer centers (CCCs) in the federal state of Baden-Württemberg in southern Germany: CCC Freiburg, CCC Heidelberg and Clinic for Thoracic Diseases Heidelberg, CCC Tübingen, and CCC Ulm.

We report this study according to the Strengthening the Reporting of Observational Studies in Epidemiology (STROBE) guideline for cross-sectional studies (see Online Resource 1) [9].

Approval was obtained from the ethics committee of the University of Freiburg. The procedures used in this study adhere to the tenets of the Declaration of Helsinki.

### Participants

Adult patients (≥ 18 years) with an initial diagnosis of advanced incurable cancer (ICD 10 C 00–80 plus ICD 10 C 78–79) diagnosed within the preceding 8 weeks were included. Cancer patients with an estimated prognosis of less than 1 year according to the physicians’ judgment using the “surprise question” were also included [10]. We excluded patients with malignant hemato-oncological diseases, dementia, psychosis/delirium, major depression, and patients who were already treated by a palliative care team.

### Recruitment and data collection

After approval of the ethics committee, patients were screened by PC physicians and nurses between July 2015 and July 2017. Patients were recruited with patient lists provided by the cancer centers, direct patient referral from oncology colleagues, participation in tumor board meetings, and by means of an electronic tool (one center) in order to ensure a representative sample. After obtaining informed consent from eligible patients, the questionnaires (see below) were answered face-to-face or sent by post.

### Outcomes

To capture a broad range of physical and psychosocial symptoms and QoL domains, a variety of validated assessment tools were used. Patients’ QoL and symptoms were assessed with the Palliative Outcome Scale [11] (POS, range 0 to 40, higher values = higher burden), the Hospital Anxiety and Depression Scale [12, 13] (HADS, range 0 to 21, higher values = higher burden; 0 to 7 = normal; 8 to 10 = mild; 11 to 14 = moderate; 15 to 21 = severe), and the European Organization for Research and Treatment of Cancer Quality of Life Questionnaire-C30 [14] (EORTC QLQ-C30, higher values = better status for Global health status/QoL and functional scales; higher values = higher burden for symptom scales/items). Patients’ needs were assessed with the POS free text by asking the patients to state their most relevant problem in the preceding 3 days. The POS was related to the preceding 3 days, the HADS and the EORTC QLQ-C30 to the preceding week.

### Cut-off scores

Validated or scale-based cut-off scores were used to define symptoms of *severe* intensity or *very low* scores for each QoL domain. Throughout the manuscript, *severe* intensity or *very low* scores are often referred to as “severe burden” and were present if at least one of the following criteria was applied:

- POS > 30
- HADS Anxiety *or* Depression score 15 to 21 (“severe”)
- Global health status/QoL item, physical functioning, role functioning, emotional functioning, cognitive functioning, *or* social functioning of the EORTC QLQ-C30 0 to 25
- Fatigue *or* nausea/vomiting of the EORTC QLQ-C30 76 to 100
- Maximum burden (= 100) in the symptoms pain, dyspnea, insomnia, appetite loss, constipation, diarrhea, *or* financial problems of the EORTC QLQ-C30.

### Data analysis

Normally distributed data are presented with means and standard deviations (SDs). Otherwise, medians and quartiles were calculated. The cut-off scores mentioned above were used to identify the number of patients with severe symptom intensity and very low scores in QoL domains, and to analyze the frequency of these symptoms and QoL domains.

Subgroup analyses included tumor site (gastrointestinal vs. respiratory system vs. genitourinary vs. breast vs. central nervous system), gender, age (< 65 vs. ≥ 65 years), and marital status (single vs. married vs. divorced vs. widowed).

The answers of the POS free text were analyzed in an inductive way by summarizing the single answers to meaningful, more general categories, e.g., pain and dyspnea were summarized into the category *physical symptoms*.

We applied descriptive statistics and analyses of variance, *t* tests, Kruskall-Wallis tests, or chi-squared tests as exploratory tests using *R* (RStudio Version 3.4.2) [15, 16]. A *p* value of < 0.05 was considered statistically significant. All statistical tests were two-sided.

## Results

**Fig. 1:**
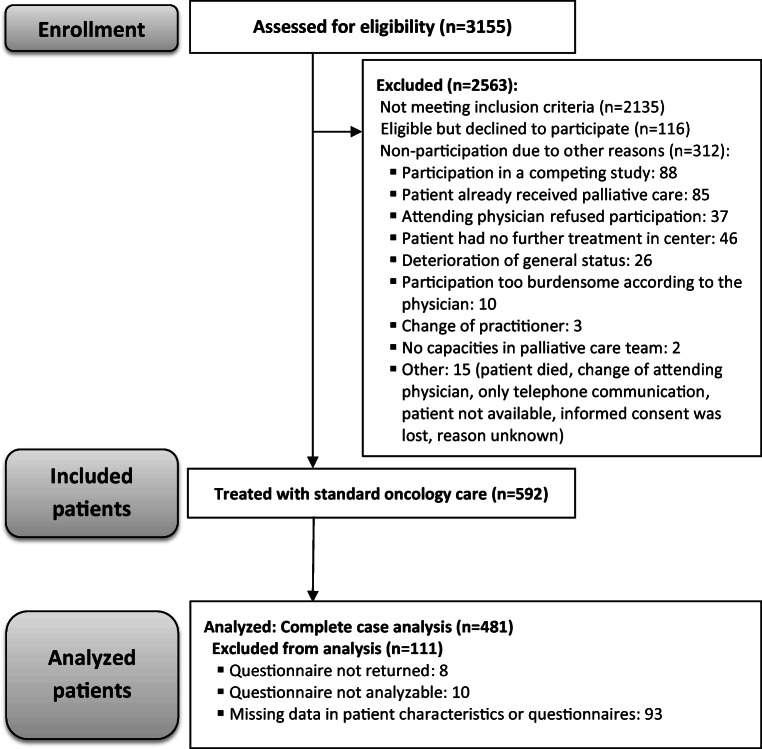
Flow diagram

Of 3155 patients screened, 592 were included (see Fig. 1) and complete data was obtained from 481 patients, 111 (18.8%) had to be excluded because the questionnaire was not returned or not assessable for other reasons (e.g., missing data).

The average age was 62.4 years (SD 12.0) and 245 (50.9%) were female. Gastrointestinal cancer (28.5%) and cancer of the respiratory system (26.6%) were the most frequent cancer diagnoses (Table 1). Average scores of POS, HADS, QoL, and symptoms were rather low or moderate. However, role functioning was the most affected functioning item (median 33.3, interquartile range (IQR) 0.0 to 66.7) and fatigue the most burdening symptom (median 55.6, IQR 33.3 to 77.8) in the EORTC QLQ-C30 questionnaire.

**Table 1:** Patient characteristics

Characteristics	Total *N* = 481
Age in years, mean (SD)	62.4 (12.0)
Sex	
Female	245 (50.9%)
Male	236 (49.1%)
Marital status	
Single	39 (8.2%)
Married	335 (70.5%)
Divorced	54 (11.4%)
Widowed	47 (9.9%)
Highest graduation (total years in school)	
General secondary school (8 years)	208 (44.3%)
Secondary school (10 years)	114 (24.3%)
High school (12 years)	45 (9.6%)
High school (13 years)	90 (19.1%)
Miscellaneous	13 (2.8%)
Tumor site	
Gastrointestinal	136 (28.5%)
Respiratory system	127 (26.6%)
Breast	40 (8.4%)
Genitourinary	70 (14.6%)
Central nervous system	33 (6.9%)
Miscellaneous	72 (15.1%)
POS score, mean (SD)	11.1 (6.4)
HADS Anxiety score, mean (SD)	6.7 (4.2)
HADS Depression score, mean (SD)	6.5 (4.7)
EORTC QLQ C30	
Global health status/QoL, mean (SD)	50.6 (23.9)
Functional scales, median (Q1, Q3)	
Physical functioning	60.0 (33.3, 83.3)
Role functioning	33.3 (0.0, 66.7)
Emotional functioning	58.3 (33.3, 75.0)
Cognitive functioning	83.3 (50.0, 100.0)
Social functioning	50.0 (16.7, 83.3)
Symptom scales, median (Q1, Q3)	
Fatigue	55.6 (33.3, 77.8)
Nausea and vomiting	0.0 (0.0, 16.7)
Pain	33.3 (0.0, 66.7)
Dyspnea	33.3 (0.0, 66.7)
Insomnia	33.3 (0.0, 66.7)
Appetite loss	33.3 (0.0, 66.7)
Constipation	0.0 (0.0, 33.3)
Diarrhea	0.0 (0.0, 33.3)
Financial difficulties	0.0 (0.0, 33.3)

Marital status: *N* = 475, 6 missing values; highest graduation: *N* = 470, 11 missing values; tumor site: *N* = 478, 3 missing values

*SD*, standard deviation; *Q1*, 1. quartile; *Q3*, 3. quartile; *QoL*, quality of life

*POS*, palliative outcome scale (range 0–40, higher values = higher burden)

*HADS*, Hospital Anxiety and Depression Scale (range 0–21; higher values = higher burden; abnormal = 11–21)

*EORTC QLQ C30*, European Organization for Research and Treatment of Cancer Quality of Life Questionnaire (higher values = better status for global health status/QoL and functional scales; higher values = higher burden for symptom scales/items)

Severe burden: at least one of the following: POS > 30; HADS A/D: severe; QLQ: quartile of most burdened patients in QoL item, physical, role, emotional, cognitive and social functioning, fatigue, and nausea/vomiting or maximum burden in the symptoms pain, dyspnea, insomnia, appetite loss, constipation, diarrhea, financial problems

### Patients with severe symptom intensity or very low QoL

**Fig. 2:**
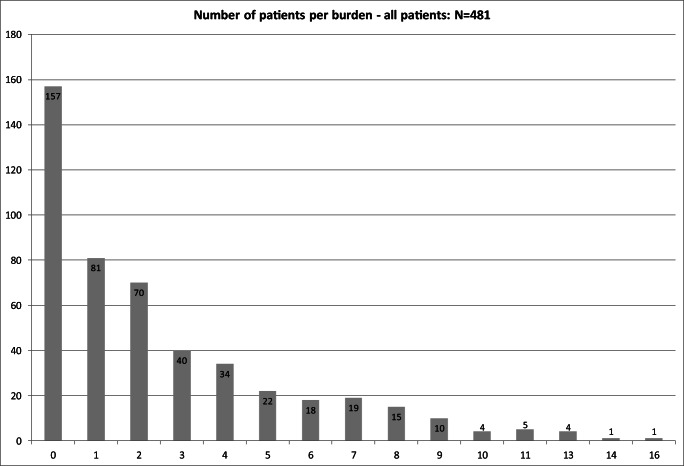
Number of patients per burden. *x*-axis, number of “severe burden”; *y*-axis, number of patients

Of 481 patients, 324 (67.4%) reported at least one symptom of *severe* intensity or *very low* QoL in at least one domain (“severe burden”), whereas 157 (32.6%) patients did not report severe burden (Fig. 2). The median number of symptoms of severe intensity or very low QoL domains per patient was 2.0 (IQR 0.0 to 4.0, range 0.0 to 16.0), and 173 (36.0%) patients suffered from three or more such “severe burdens” (Online Resource 2).

In the subgroup of patients who reported at least one severe symptom or very low scores for at least one QoL domain (*n* = 324), the median number of such “severe burdens” was 3.0 per patient (IQR 1.8 to 5.0, range 1.0 to 16) (Online Resource 3).

### Symptoms and QoL domains that were most often severely affected

**Fig. 3:**
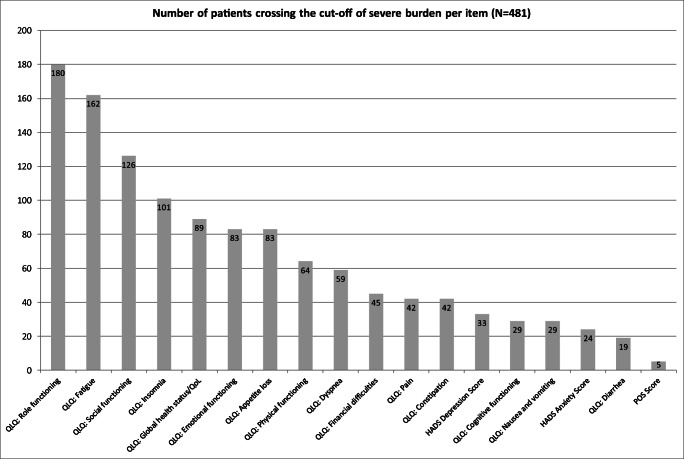
Number of patients crossing the cut-off of severe burden per item. Patients with at least one severe burden were defined according to the following criteria: POS > 30 or HADS Anxiety or Depression score 15 to 21 (“severe”) or global health status/quality of life-item, physical functioning, role functioning, emotional functioning, cognitive functioning, or social functioning of the EORTC QLQ-C30 0 to 25 or fatigue or nausea/vomiting of the EORTC QLQ-C30 76 to 100 or maximum burden (= 100) in the symptoms pain, dyspnea, insomnia, appetite loss, constipation, diarrhea, or financial problems of the EORTC QLQ-C30

Figure 3 shows the number of patients crossing the cut-off of “severe burden” per item. Role functioning (*n* = 180, 37.4%), fatigue (*n* = 162, 33.7%), and social functioning (*n* = 126, 26.2%) were the three most common reasons for severe burden. QoL was considered to cause severe burden in 89 patients (18.5%) and depressive symptoms in 33 patients (6.9%). Dyspnea led to severe burden in 59 patients (12.3%) and pain in 42 patients (8.7%).

The HADS Anxiety score was normal in 292 (60.7%), mild in 104 (21.6%), moderate in 61 (12.7%), and severe in 24 (5.0%) patients. The HADS Depression score was normal in 314 (65.3%), mild in 69 (14.3%), moderate in 65 (13.5%), and severe in 33 (6.9%) patients.

According to the POS free text, the most relevant problem in the past 3 days were (i) physical symptoms (*n* = 93, 26.8%), followed by (ii) fear from adverse events (*n* = 32, 9.2%), and (iii) disease and death, dealing with illness (30, 8.7%) (see Table 2).

**Table 2:** POS free text

Most relevant problem in the past 3 days	Total: *N* = 347
Physical symptoms (pain, dyspnea, etc.)	93 (26.8%)
Fear from adverse events (e.g., chemotherapy, radiation)	32 (9.2%)
Disease and death, dealing with illness	30 (8.7%)
Life expectancy, prognosis, chances for cure/relief	28 (8.1%)
Financial concerns	23 (6.6%)
No problems	21 (6.1%)
Miscellaneous problems	20 (5.8%)
Therapeutic decision	19 (5.5%)
Concerns about family and relatives	17 (4.9%)
Home care, autonomy	15 (4.3%)
Uncertainty and concerns about future	14 (4.0%)
Psychological burden (depression, anxiety)	12 (3.5%)
Organization (e.g., transport to hospital, scheduling)	11 (3.2%)
Concern about work	9 (2.6%)
Reasons of disease	3 (0.9%)

Problems listed in descending order according to the total number and percentage

Percentages refer to number in column

*POS*, Palliative Outcome Scale

### Subgroup analysis

Tables regarding the exploratory subgroup analyses for tumor site, gender, age, and marital status can be found in Online Resources 4 to 7.

Patients with cancer in the respiratory system reported higher impairment on the POS (12.1, SD 6.3) and HADS Depression score (7.3, SD 4.8), higher intensity of insomnia (median 66.7), and lower QoL (45.7, SD 23.3) measured with the EORTC QLQ-C30 global health status/QoL item compared to the other tumor sites. Patients with central nervous system tumors had low values in physical, role, and social functioning (medians 46.7, 16.7, and 33.3, respectively). Pain was most present in patients with genitourinary cancer (median 50.0). The absolute differences between patients of tumor entities were rather small (Online Resource 4).

The subgroup analysis for gender showed that women reported significantly higher POS scores (11.7, SD 6.6 vs. 10.5, SD 6.1), HADS Anxiety scores (7.2, SD 4.3 vs. 6.2, SD 4.0), higher levels of fatigue (medians 66.7 vs. 55.6), and slightly lower values in the emotional functioning item (Online Resource 5).

Dividing the sample according to the often used threshold < 65 vs. ≥ 65 years yielded groups that were on average 54.0 years (SD 8.1) and 73.3 years (SD 6.0) old. Patients ≥ 65 years reported lower values on the physical function scale (medians 53.3 vs. 66.7) and fewer financial difficulties (medians 0.0 vs. 33.3) (Online Resource 6).

Marital status was hardly associated with outcomes except for some variations in financial difficulties, with divorced patients perceived as having the highest financial burden of the groups (median 33.3) (Online Resource 7).

## Discussion

Rather than focusing on mean intensity of symptoms, the aims of this the study were to identify (i) how many newly diagnosed outpatients with advanced cancer suffered from symptoms of *severe intensity* and *very low* QoL domains and (ii) which symptoms and QoL domains were the most frequent causes for such suffering.

The mean scores for QoL and symptom intensity were generally rather low or moderate, which was comparable to some previous early palliative care (EPC) trials [17–20]. In the present study, we focused on symptoms of severe intensity or QoL domains that were reported to be very low by the patients. Two-thirds of the patients experienced at least one of such “severe burdens” with a median of two per patient. Role functioning, fatigue, and social functioning were identified as the three most common reasons for such “severe burdens” already shortly after the diagnosis of incurable advanced cancer, with scores being clearly worse than in the normative population [21]. In studies with palliative care patients, fatigue, pain, weakness, appetite loss, anorexia, constipation, anxiety, and depression were identified as burdening symptoms, but in general, only means and SDs are reported in such trials [2, 3, 6, 22–24]. Functioning items were mostly not assessed in these previous studies except for the study by Lidstone et al. in which cancer outpatients rated their symptoms and concerns [22]. Similar to our results from patients shortly after diagnosis, patients from the study of Lidstone et al. reported role functioning to be problematic as addressed by the items “Not being able to do the things you usually do” and “Relationships with important people in your life” [22].

Our analysis also revealed that one-fifth of the newly diagnosed advanced cancer patients experienced moderate or severe anxiety and depression symptoms and a very low QoL. While normative data for the HADS Anxiety score suggest almost equal distribution compared to the normative population [25], the normative data for the HADS Depression score [25] and the EORTC QLQ-C30 [21, 26] revealed that depression and impairment of QoL was pronounced already in the patients early after diagnosis of advanced cancer in the study at hand.

In the free text of the POS, patients often reported fears from adverse events, e.g., from tumor-directed therapy or the planned diagnostic procedures, which can substantially aggravate depression and anxiety as well as impair patients’ QoL. This finding is important to consider when planning integrated specialist palliative care and oncology cooperations because for providing help for coping with these fears, the *basic*, *primary*, or *general* palliative care expertise of the oncology team is needed to address these specific information needs, to initiate goal-of-care discussions, or to overcome misunderstandings concerning therapeutic or diagnostic maneuvers. Such integration of *general* palliative care provided by the oncology team is often overlooked in the conceptual development of oncology/specialist palliative care cooperations for EPC [1, 27].

Online Resources 4 and 5 indicated that tumor site and gender might be associated with patients’ burden. In all, women had better POS scores but reported higher values for anxiety and fatigue than male patients which confirms results identified in other studies and underlines the need for a closer look at gender differences in future studies [28–30].

Most notably, women with breast cancer tended to have better physical and emotional functioning and less fatigue compared to other female cancer patients. The reason for this finding is unclear, but it may be argued, that because of an exceptional long-standing tradition of interdisciplinary, multi-professional, comprehensive breast cancer treatment tradition, these findings may illustrate the potential merits of multi-professional and interdisciplinary comprehensive cancer care.

Patients with central nervous system tumors had a more severely impaired physical, role, and social functioning whereas patients with tumors in the respiratory system reported rather high intensity of symptoms regarding insomnia, yet better values in the POS score.

The importance of screening and identification of unmet symptoms and needs and the subsequent integration of specialist palliative care have been emphasized and may result in better patient-relevant outcomes [31]. The large percentage of severely affected advanced cancer patients with a recent advanced cancer diagnosis yielded in this study adds to the discussion of how burdened patients should be identified [32–34].

In a Delphi study with nearly 60 international experts, 11 criteria concerning a possible outpatient palliative care referral were found in a consensus process [34]. The criteria involve physical, emotional, and spiritual dimensions. They also include assistance with decision making, patient request, as well as two time-based criteria [34]. These criteria are in accordance with the findings in this study, emphasizing that newly diagnosed advance cancer patients may suffer in the physical, psychological, and social dimensions.

The referral process remains challenging and may depend on hospital structures [1]. Experts agreed in a recent Delphi study with an 86% agreement rate that a combination of both automatic and clinician-based referral may be a meaningful solution [35]. The data presented in this study could serve to discuss and test various concepts for screening models to identify burdening symptoms and patients’ needs.

### Limitations

In accordance with the real-world design of the study, we used different recruitment models within and between the centers: lists, tumor board meetings, electronic tool, and direct referral. The varying recruitment models reflect the challenges of identifying patients early after diagnosis of advanced cancer and we suspect that not all potentially eligible patients were screened with this approach.

Another limitation was the time variation from diagnosis to study inclusion between patients due to the outpatient setting, although it usually did not exceed a time frame of 8 weeks. This could have had an impact on the symptom burden of the patients and contributed to the variability of the sample.

Reporting moderate or severe symptoms does not automatically mean that the patients also perceived a need for treatment. Therefore, we refer to needs only when reporting or discussing the results of the POS free text in which the patients were asked for their most relevant problem in the preceding 3 days.

Due to the small sample sizes and the exploratory character of the subgroup analyses, conclusions based on these data should be drawn carefully.

Finally, our definition of patients with at least one “severe burden” for the POS score and the EORTC QLQ-C30, which represented a low QoL, low functioning, or high symptom burden, was scale-based (e.g., POS > 30, POS range 0 to 40), which is not validated yet. Nevertheless, the cut-off for defining severe burden for the HADS (15–21 = severe) is validated and well-accepted [12, 13].

### Implications for practice

This data contributes to a better understanding of the QoL and symptom burden of newly diagnosed advanced cancer patients, and suggests tumor site and gender as factors that may be considered in screening processes and in the treatment of patients. Potential screening methods can be discussed and tested based on the present data. In this sense, the data underlines the high importance of EPC, the necessity of screening programs, and the inclusion of a multidisciplinary specialist EPC team.

## Conclusion

Two-thirds of patients experienced at least one severe symptom or a very low QoL within the first 8 weeks after diagnosis of advanced cancer with role functioning, fatigue, and social functioning being the three most common reasons for “severe burden” in this EPC population. Tumor site and gender may be considered as additional factors for early routine assessment of patient burden. In light of these findings, the necessity of screening programs and the integration of oncology and a multiprofessional specialist EPC team gains importance to provide high-quality treatment of newly diagnosed advanced cancer patients.

## Electronic supplementary material

ESM 1 (DOCX 154 kb).
