# Supplementary material for: Severe symptoms and very low quality-of-life among outpatients newly diagnosed with advanced cancer: data from a multicenter cohort study
Source: Support Care Cancer. 2020 Mar 17;28(11):5547–55. doi: 10.1007/s00520-020-05388-y (PMC7547028; doi:10.1007/s00520-020-05388-y)
Supplement: Supplementary file 1 — (DOCX 154 kb). [file 520_2020_5388_MOESM1_ESM.docx]

**Journal: Supportive Care in Cancer**

**Severe symptoms and very low quality-of-life among outpatients newly diagnosed with advanced cancer. Data from a multicenter cohort study**

Authors: Waldemar Siemens^1^ (MSc), Stefan S. Schönsteiner^2^ (MD), Claudia Lorena Orellana-Rios^1^ (MSc), Ulrike Schaekel^3^ (MD), Jens Kessler^4^ (Prof), Corinna Eschbach^5^ (MD), Marén Viehrig^6^ (MD), Regine Mayer-Steinacker^2^ (MD), Gerhild Becker^1^* (Prof), Jan Gaertner^7^* (MD) _* contributed equally._

^1^Clinic for Palliative Care, Medical Centre, University of Freiburg, Faculty of Medicine, University of Freiburg, Freiburg, Germany

^2^University Hospital Ulm, Department of Internal Medicine III, Ulm, Germany

^3^Heidelberg University Hospital, Internal Medicine V, Hematology/Oncology/Rheumatology, Heidelberg, Germany

^4^University Hospital Heidelberg, Centre of Pain Therapy and Palliative Care Medicine, Department of Anesthesiology, Heidelberg, Germany

^5^University Hospital Heidelberg and Translational Lung Research Centre Heidelberg (TLRC-H), Department of Thoracic Oncology, Member of the German Centre for Lung Research (DZL), Heidelberg, Germany

^6^University Hospital of Tübingen, Department of Radiooncology, Palliative Care Unit, Tuebingen, Germany

^7^Center for Palliative Care Hildegard, Basel, Switzerland

**Corresponding author:** Waldemar Siemens

Clinic for Palliative Care, Medical Centre, University of Freiburg,

Robert-Koch-Str 3, 79106 Freiburg, Germany

Phone: Tel: +49 761 270-95418

Email: [waldemar.siemens@googlemail.com](mailto:waldemar.siemens@googlemail.com)

**Online Resources**

[Online Resource 1: STROBE Statement – Checklist for cross-sectional studies 3](#_Toc31114423)

[Online Resource 2: Number of patients per burden – all patients 5](#_Toc31114424)

[Online Resource 3: Number of patients per burden – patients with severe burden 6](#_Toc31114425)

[Online Resource 4: Subgroup analysis: tumor site 10](#_Toc31114426)

[Online Resource 5: Subgroup analysis: gender 16](#_Toc31114427)

[Online Resource 6: Subgroup analysis: age 20](#_Toc31114428)

[Online Resource 7: Subgroup analysis: marital status 24](#_Toc31114429)

# Online Resource 1: STROBE Statement – Checklist for cross-sectional studies

|  | Item No | Recommendation | Page |
| --- | --- | --- | --- |
| **Title and abstract** | 1 | (*a*) Indicate the study’s design with a commonly used term in the title or the abstract | 1 |
|  |  | (*b*) Provide in the abstract an informative and balanced summary of what was done and what was found | 1 |
| Introduction | | |  |
| Background/rationale | 2 | Explain the scientific background and rationale for the investigation being reported | 2 |
| Objectives | 3 | State specific objectives, including any prespecified hypotheses | 2 |
| Methods | | |  |
| Study design | 4 | Present key elements of study design early in the paper | 2 |
| Setting | 5 | Describe the setting, locations, and relevant dates, including periods of recruitment, exposure, follow-up, and data collection | 2 |
| Participants | 6 | (*a*) Give the eligibility criteria, and the sources and methods of selection of participants | 2 |
| Variables | 7 | Clearly define all outcomes, exposures, predictors, potential confounders, and effect modifiers. Give diagnostic criteria, if applicable | 2 |
| Data sources/ measurement | 8* | For each variable of interest, give sources of data and details of methods of assessment (measurement). Describe comparability of assessment methods if there is more than one group | 2 |
| Bias | 9 | Describe any efforts to address potential sources of bias | 2 |
| Study size | 10 | Explain how the study size was arrived at | n.a. |
| Quantitative variables | 11 | Explain how quantitative variables were handled in the analyses. If applicable, describe which groupings were chosen and why | 2 |
| Statistical methods | 12 | (*a*) Describe all statistical methods, including those used to control for confounding | 3 |
|  |  | (*b*) Describe any methods used to examine subgroups and interactions | 3 |
|  |  | (*c*) Explain how missing data were addressed | 3 |
|  |  | (*d*) If applicable, describe analytical methods taking account of sampling strategy | n.a. |
|  |  | (*e*) Describe any sensitivity analyses | n.a. |
| Results | | |  |
| Participants | 13* | (a) Report numbers of individuals at each stage of study—eg numbers potentially eligible, examined for eligibility, confirmed eligible, included in the study, completing follow-up, and analysed | 3,  Figure 1 |
|  |  | (b) Give reasons for non-participation at each stage | 3,  Figure 1 |
|  |  | (c) Consider use of a flow diagram | Figure 1 |
| Descriptive data | 14* | (a) Give characteristics of study participants (eg demographic, clinical, social) and information on exposures and potential confounders | 3, Table 1 |
|  |  | (b) Indicate number of participants with missing data for each variable of interest | n.a. |
| Outcome data | 15* | Report numbers of outcome events or summary measures | 3, 4 |
| Main results | 16 | (*a*) Give unadjusted estimates and, if applicable, confounder-adjusted estimates and their precision (eg, 95% confidence interval). Make clear which confounders were adjusted for and why they were included | n.a. |
|  |  | (*b*) Report category boundaries when continuous variables were categorized | 2, 3 |
|  |  | (*c*) If relevant, consider translating estimates of relative risk into absolute risk for a meaningful time period | n.a. |
| Other analyses | 17 | Report other analyses done—eg analyses of subgroups and interactions, and sensitivity analyses | 3, 4, Figure 2 |
| Discussion | | |  |
| Key results | 18 | Summarise key results with reference to study objectives | 4 |
| Limitations | 19 | Discuss limitations of the study, taking into account sources of potential bias or imprecision. Discuss both direction and magnitude of any potential bias | 6 |
| Interpretation | 20 | Give a cautious overall interpretation of results considering objectives, limitations, multiplicity of analyses, results from similar studies, and other relevant evidence | 4-7 |
| Generalisability | 21 | Discuss the generalisability (external validity) of the study results | 6 |
| Other information | | |  |
| Funding | 22 | Give the source of funding and the role of the funders for the present study and, if applicable, for the original study on which the present article is based | 8 |

*Give information separately for exposed and unexposed groups.

n.a.: not applicable; STROBE: Strengthening the Reporting of Observational Studies in Epidemiology guideline

**Note:** An Explanation and Elaboration article discusses each checklist item and gives methodological background and published examples of transparent reporting. The STROBE checklist is best used in conjunction with this article (freely available on the Web sites of PLoS Medicine at http://www.plosmedicine.org/, Annals of Internal Medicine at http://www.annals.org/, and Epidemiology at http://www.epidem.com/). Information on the STROBE Initiative is available at www.strobe-statement.org.

# Online Resource 2: Number of patients per burden – all patients

**Number of patients per burden – all patients: N=481**

| **Burden** | **0** | **1** | **2** | **3** | **4** | **5** | **6** | **7** | **8** | **9** | **10** | **11** | **12** | **13** | **14** | **15** | **16** |
| --- | --- | --- | --- | --- | --- | --- | --- | --- | --- | --- | --- | --- | --- | --- | --- | --- | --- |
| **Patients** | 157 | 81 | 70 | 40 | 34 | 22 | 18 | 19 | 15 | 10 | 4 | 5 | 0 | 4 | 1 | 0 | 1 |

**Number of patients per burden – all patients: N=481 – Summary statistics**

| **Minimum** | **1st Quartile** | **Median** | **Mean** | **3rd Quartile** | **Maximum** |
| --- | --- | --- | --- | --- | --- |
| 0.0 | 0.0 | 2.0 | 2.5 | 4.0 | 16.0 |

x-axis: number of “severe burden”; y-axis: number of patients

# Online Resource 3: Number of patients per burden – patients with severe burden

**Number of patients per burden – subset of patients with severe burden: N=324**

| **Burden** | **0** | **1** | **2** | **3** | **4** | **5** | **6** | **7** | **8** | **9** | **10** | **11** | **12** | **13** | **14** | **15** | **16** |
| --- | --- | --- | --- | --- | --- | --- | --- | --- | --- | --- | --- | --- | --- | --- | --- | --- | --- |
| **Patients** | 0 | 81 | 70 | 40 | 34 | 22 | 18 | 19 | 15 | 10 | 4 | 5 | 0 | 4 | 1 | 0 | 1 |

**Number of patients per burden – subset of patients with severe burden: N=324 – Summary statistics**

| **Minimum** | **1st Quartile** | **Median** | **Mean** | **3rd Quartile** | **Maximum** |
| --- | --- | --- | --- | --- | --- |
| 1.0 | 1.8 | 3.0 | 3.8 | 5.0 | 16.0 |

x-axis: number of “severe burden”; y-axis: number of patients

**POS, HADS and EORTC QLQ-C30 in proportions by group, and total**

| **Outcomes in proportions^$^** | **Control**  **N=273** | **Intervention**  **N=208** | **Total**  **N=481** |
| --- | --- | --- | --- |
| **POS Score** |  |  |  |
| 0-10 | 135 (49.5%) | 120 (57.7%) | 255 (53.0%) |
| 11-20 | 114 (41.8%) | 71 (34.1%) | 185 (38.5%) |
| 21-30 | 21 (7.7%) | 15 (7.2%) | 36 (7.5%) |
| 31-40 | 3 (1.1%) | 2 (1.0%) | 5 (1.0%) |
| **HADS Anxiety Score** |  |  |  |
| Normal=0-7 | 164 (60.1%) | 128 (61.5%) | 292 (60.7%) |
| Mild=8-10 | 58 (21.2%) | 46 (22.1%) | 104 (21.6%) |
| Moderate=11-14 | 36 (13.2%) | 25 (12.0%) | 61 (12.7%) |
| Severe=15-21 | 15 (5.5%) | 9 (4.3%) | 24 (5.0%) |
| **HADS Depression Score** |  |  |  |
| Normal=0-7 | 170 (62.3%) | 144 (69.2%) | 314 (65.3%) |
| Mild=8-10 | 44 (16.1%) | 25 (12.0%) | 69 (14.3%) |
| Moderate=11-14 | 40 (14.7%) | 25 (12.0%) | 65 (13.5%) |
| Severe=15-21 | 19 (7.0%) | 14 (6.7%) | 33 (6.9%) |
| **EORTC QLQ C30** |  |  |  |
| **Global health status/QoL** |  |  |  |
| 0-25 | 55 (20.1%) | 34 (16.3%) | 89 (18.5%) |
| 26-50 | 107 (39.2%) | 66 (31.7%) | 175 (36.0%) |
| 51-75 | 79 (28.9%) | 79 (38.0%) | 158 (32.8%) |
| 76-100 | 32 (11.7%) | 29 (13.9%) | 61 (12.7%) |
| **Functional scales** |  |  |  |
| Physical functioning |  |  |  |
| 0-25 | 32 (11.7%) | 32 (15.4%) | 64 (13.3%) |
| 26-50 | 73 (26.7%) | 36 (17.3%) | 109 (22.7%) |
| 51-75 | 89 (32.6%) | 69 (33.2%) | 158 (32.8%) |
| 76-100 | 79 (28.9%) | 71 (34.1%) | 150 (31.2%) |
| Role functioning |  |  |  |
| 0-25 | 104 (38.1%) | 76 (36.5%) | 180 (37.4%) |
| 26-50 | 73 (26.7%) | 46 (22.1%) | 119 (24.7%) |
| 51-75 | 45 (16.5%) | 40 (19.2%) | 85 (17.7%) |
| 76-100 | 51 (18.7%) | 46 (22.1%) | 97 (20.2%) |
| Emotional functioning |  |  |  |
| 0-25 | 56 (20.5%) | 27 (13.0%) | 83 (17.3%) |
| 26-50 | 72 (26.4%) | 52 (25.0%) | 124 (25.8%) |
| 51-75 | 87 (31.9%) | 72 (34.6%) | 159 (33.1%) |
| 76-100 | 58 (21.2%) | 57 (27.4%) | 115 (23.9%) |
| Cognitive functioning |  |  |  |
| 0-25 | 14 (5.1%) | 15 (7.2%) | 29 (6.0%) |
| 26-50 | 62 (22.7%) | 37 (17.8%) | 99 (20.6%) |
| 51-75 | 48 (17.6%) | 34 (16.3%) | 82 (17.0%) |
| 76-100 | 149 (54.6%) | 122 (58.7%) | 271 (56.3%) |
| Social functioning |  |  |  |
| 0-25 | 74 (27.1%) | 52 (25.0%) | 126 (26.2%) |
| 26-50 | 81 (29.7%) | 66 (31.7%) | 147 (30.6%) |
| 51-75 | 48 (17.6%) | 33 (15.9%) | 81 (16.8%) |
| 76-100 | 70 (25.6%) | 57 (27.4%) | 127 (26.4%) |
| **Symptom scales** |  |  |  |
| Fatigue |  |  |  |
| 0-25 | 48 (17.6%) | 37 (17.8%) | 85 (17.7%) |
| 26-50 | 64 (23.4%) | 55 (26.4%) | 119 (24.7%) |
| 51-75 | 67 (24.5%) | 48 (23.1%) | 115 (23.9%) |
| 76-100 | 94 (34.4%) | 68 (32.7%) | 162 (33.7%) |
| Nausea and vomiting |  |  |  |
| 0-25 | 203 (74.4%) | 158 (76.0%) | 361 (75.1%) |
| 26-50 | 50 (18.3%) | 30 (14.4%) | 80 (16.6%) |
| 51-75 | 6 (2.2%) | 5 (2.4%) | 11 (2.3%) |
| 76-100 | 14 (5.1%) | 15 (7.2%) | 29 (6.0%) |
| Pain |  |  |  |
| 0 | 104 (38.1%) | 98 (47.1%) | 202 (42.0%) |
| 33.3 | 75 (27.5%) | 49 (23.6%) | 124 (25.8%) |
| 66.6 | 41 (15.0%) | 26 (12.5%) | 67 (13.9%) |
| 100 | 53 (19.4%) | 35 (16.8%) | 88 (18.3%) |
| Dyspnea |  |  |  |
| 0 | 119 (43.6%) | 95 (45.7%) | 214 (44.5%) |
| 33.3 | 67 (24.5%) | 53 (25.5%) | 120 (24.9%) |
| 66.6 | 45 (16.5%) | 43 (20.7%) | 88 (18.3%) |
| 100 | 42 (15.4%) | 17 (8.2%) | 59 (12.3%) |
| Insomnia |  |  |  |
| 0 | 82 (30.0%) | 77 (37.0%) | 159 (33.1%) |
| 33.3 | 63 (23.1%) | 46 (22.1%) | 109 (22.7%) |
| 66.6 | 63 (23.1%) | 49 (23.6%) | 112 (23.3%) |
| 100 | 65 (23.8%) | 36 (17.3%) | 101 (21.0%) |
| Appetite loss |  |  |  |
| 0 | 132 (48.4%) | 104 (50.0%) | 236 (49.1%) |
| 33.3 | 50 (18.3%) | 36 (17.3%) | 86 (17.9%) |
| 66.6 | 48 (17.6%) | 28 (13.5%) | 76 (15.8%) |
| 100 | 43 (15.8%) | 40 (19.2%) | 83 (17.3%) |
| Constipation |  |  |  |
| 0 | 161 (59.0%) | 127 (61.1%) | 288 (59.9%) |
| 33.3 | 47 (17.2%) | 28 (13.5%) | 75 (15.6%) |
| 66.6 | 41 (15.0%) | 35 (16.8%) | 76 (15.8%) |
| 100 | 24 (8.8%) | 18 (8.7%) | 42 (8.7%) |
| Diarrhea |  |  |  |
| 0 | 201 (73.6%) | 150 (72.1%) | 351 (73.0%) |
| 33.3 | 36 (13.2%) | 31 (14.9%) | 67 (13.9%) |
| 66.6 | 26 (9.5%) | 18 (8.7%) | 44 (9.1%) |
| 100 | 10 (3.7%) | 9 (4.3%) | 19 (4.0%) |
| Financial difficulties |  |  |  |
| 0 | 160 (58.6%) | 114 (54.8%) | 274 (57.0%) |
| 33.3 | 50 (18.3%) | 40 (19.2%) | 90 (18.7%) |
| 66.6 | 42 (15.4%) | 30 (14.4%) | 72 (15.0%) |
| 100 | 21 (7.7%) | 24 (11.5%) | 45 (9.4%) |

* Chi-squared-tests for patients in control and intervention group: p<0.05

^$^ POS and EORTC QLQ-C30 in four equal proportions: Global health status/QoL, functional scales and fatigue; other symptom scales of EORTC QLQ-C30: only four values: 0, 33.3, 66.6, 100; HADS according to clinical classification

POS: Palliative Outcome Scale (range: 0-40, higher values = higher burden)

HADS: Hospital Anxiety and Depression Scale (range: 0-21; higher values = higher burden)

EORTC QLQ C30: European Organization for Research and Treatment of Cancer Quality of Life Questionnaire (higher values = better status for Global health status/QoL and functional scales; higher values = higher burden for symptom scales / items)

**POS free text by group, and total**

| **Most relevant problem in the past three days** | **Control**  **N=195** | **Intervention**  **N=152** | **Total**  **N=347** |
| --- | --- | --- | --- |
| Physical symptoms (pain, dyspnea etc.) | 45 (23.1%) | 48 (31.6%) | 93 (26.8%) |
| Fear from adverse events (e.g. chemotherapy, radiation) | 21 (10.8%) | 11 (7.2%) | 32 (9.2%) |
| Disease and death, dealing with illness | 19 (9.7%) | 11 (7.2%) | 30 (8.7%) |
| Life expectancy, prognosis, chances for cure / relief | 21 (10.8%) | 7 (4.6%) | 28 (8.1%) |
| Financial concerns | 13 (6.7%) | 10 (6.6%) | 23 (6.6%) |
| No problems | 12 (6.2%) | 9 (5.9%) | 21 (6.1%) |
| Miscellaneous problems | 6 (3.1%) | 14 (9.2%) | 20 (5.8%) |
| Therapeutic decision | 8 (4.1%) | 11 (7.2%) | 19 (5.5%) |
| Concerns about family and relatives | 7 (3.6%) | 10 (6.6%) | 17 (4.9%) |
| Home care, autonomy | 11 (5.6%) | 4 (2.6%) | 15 (4.3%) |
| Uncertainty and concerns about future | 9 (4.6%) | 5 (3.3%) | 14 (4.0%) |
| Psychological burden (depression, anxiety) | 10 (5.1%) | 2 (1.3%) | 12 (3.5%) |
| Organization (e.g. transport to hospital, scheduling) | 5 (2.6%) | 6 (4.0%) | 11 (3.2%) |
| Concern about work | 5 (2.6%) | 4 (2.6%) | 9 (2.6%) |
| Reasons of disease | 3 (1.5%) | 0 (0.0%) | 3 (0.9%) |

Problems listed in descending order according to the total number and percent

Percentages refer to number in column

# Online Resource 4: Subgroup analysis: tumor site

**Patient characteristics by tumor site, and total**

| **Characteristics** | **Gastrointestinal**  **N=136** | **Respiratory system**  **N=127** | **Genitourinary**  **N=70** | **Breast**  **N=40** | **Central nervous system, N=33** | **Total**  **N=406^$^** |
| --- | --- | --- | --- | --- | --- | --- |
| **Age in years***, mean (SD) | 62.7 (10.5) | 63.7 (10.6) | 64.0 (12.7) | 58.1 (15.5) | 59.3 (13.3) | 62.5 (11.8) |
| **Sex*:** |  |  |  |  |  |  |
| female | 48 (35.3%) | 51 (40.2%) | 61 (87.1%) | 39 (97.5%) | 16 (48.5%) | 215 (53.0%) |
| male | 88 (64.7%) | 76 (59.8%) | 9 (12.9%) | 1 (2.5%) | 17 (51.5%) | 191 (47.0%) |
| **Marital status:** |  |  |  |  |  |  |
| single | 11 (8.3%) | 9 (7.1%) | 5 (7.14%) | 5 (12.8%) | 1 (3.1%) | 31 (7.7%) |
| married | 97 (72.9%) | 94 (74.0%) | 41 (58.6%) | 26 (66.7%) | 26 (81.2%) | 284 (70.8%) |
| divorced | 13 (9.8%) | 12 (9.6%) | 13 (18.6%) | 3 (7.69%) | 4 (12.5%) | 45 (11.2%) |
| widowed | 12 (9.0%) | 12 (9.6%) | 11 (15.7%) | 5 (12.8%) | 1 (3.12%) | 41 (10.2%) |
| **Highest graduation (total years in school):** |  |  |  |  |  |  |
| general secondary school (8 years) | 53 (39.3%) | 63 (50.8%) | 31 (44.9%) | 15 (38.5%) | 11 (35.5%) | 173 (43.5%) |
| secondary school (10 years) | 36 (26.7%) | 30 (24.2%) | 15 (21.7%) | 9 (23.1%) | 7 (22.6%) | 97 (24.4%) |
| high school (12 years) | 15 (11.1%) | 13 (10.5%) | 6 (8.70%) | 3 (7.7%) | 2 (6.5%) | 39 (9.8%) |
| high school (13 years) | 28 (20.7%) | 13 (10.5%) | 15 (21.7%) | 12 (30.8%) | 9 (29.0%) | 77 (19.3%) |
| miscellaneous | 3 (2.2%) | 5 (4.0%) | 2 (2.90%) | 0 (0.0%) | 2 (6.5%) | 12 (3.0%) |
|  |  |  |  |  |  |  |
| **POS Score***, mean (SD) | 9.9 (6.6) | 12.1 (6.3) | 11.7 (5.8) | 11.2 (6.1) | 9.6 (4.3) | 11.0 (6.2) |
| **HADS Anxiety Score**, mean (SD) | 6.2 (4.0) | 7.3 (4.1) | 6.7 (4.1) | 6.4 (4.2) | 7.0 (3.8) | 6.7 (4.1) |
| **HADS Depression Score***, mean (SD) | 5.9 (4.7) | 7.3 (4.8) | 6.4 (4.0) | 5.3 (4.0) | 7.1 (4.6) | 6.5 (4.6) |
| **EORTC QLQ C30** |  |  |  |  |  |  |
| **Global health status/QoL***, mean (SD) | 53.9 (23.6) | 45.7 (23.3) | 51.9 (22.9) | 57.5 (21.1) | 51.3 (20.3) | 51.1 (23.1) |
| **Functional scales**, median [Q_1_, Q_3_] |  |  |  |  |  |  |
| Physical functioning* | 66.7 [45.0;86.7] | 53.3 [30.0;77.5] | 60.0 [40.0;78.3] | 73.3 [46.7;86.7] | 46.7 [6.7;73.3] | 60.0 [35.0;80.0] |
| Role functioning* | 50.0 [16.7;66.7] | 33.3 [0.0;66.7] | 33.3 [16.7;66.7] | 50.0 [29.2;66.7] | 16.7 [0.0;66.7] | 33.3 [0.0;66.7] |
| Emotional functioning | 66.7 [41.7;83.3] | 58.3 [33.3;75.0] | 58.3 [33.3;75.0] | 62.5 [33.3;83.3] | 50.0 [41.7;58.3] | 58.3 [33.3;75.0] |
| Cognitive functioning* | 83.3 [66.7;100.0] | 83.3 [50.0;100.0] | 83.3 [66.7;100.0] | 83.3 [62.5;100.0] | 66.7 [33.3;83.3] | 83.3 [50.0;100.0] |
| Social functioning* | 66.7 [33.3;83.3] | 50.0 [16.7;66.7] | 50.0 [33.3;79.2] | 50.0 [33.3;83.3] | 33.3 [0.0;50.0] | 50.0 [16.7;83.3] |
| **Symptom scales**, median [Q_1_, Q_3_] |  |  |  |  |  |  |
| Fatigue | 55.6 [33.3;77.8] | 66.7 [33.3;88.9] | 66.7 [36.1;77.8] | 44.4 [41.7;66.7] | 55.6 [44.4;88.9] | 55.6 [33.3;77.8] |
| Nausea and vomiting | 0.0 [0.0;33.3] | 0.0 [0.0;33.3] | 0.0 [0.0;16.7] | 0.0 [0.0;33.3] | 0.0 [0.0;0.0] | 0.0 [0.0;33.3] |
| Pain* | 16.7 [0.0;50.0] | 33.3 [0.0;66.7] | 50.0 [16.7;66.7] | 41.7 [16.7;66.7] | 16.7 [0.0;33.3] | 33.3 [0.0;66.7] |
| Dyspnea* | 0.0 [0.0;33.3] | 33.3 [0.0;66.7] | 33.3 [0.0;66.7] | 33.3 [0.0;66.7] | 0.0 [0.0;33.3] | 33.3 [0.0;66.7] |
| Insomnia | 33.3 [0.0;66.7] | 66.7 [0.0;100.0] | 50.0 [0.0;66.7] | 33.3 [0.0;66.7] | 33.3 [0.0;66.7] | 33.3 [0.0;66.7] |
| Appetite loss* | 33.3 [0.0;66.7] | 33.3 [0.0;66.7] | 0.0 [0.0;66.7] | 33.3 [0.0;41.7] | 0.0 [0.0;33.3] | 33.3 [0.0;66.7] |
| Constipation | 0.0 [0.0;33.3] | 0.0 [0.0;33.3] | 16.7 [0.0;66.7] | 33.3 [0.0;66.7] | 0.0 [0.0;33.3] | 0.0 [0.0;33.3] |
| Diarrhea* | 0.0 [0.0;33.3] | 0.0 [0.0;0.0] | 0.0 [0.0;25.0] | 0.0 [0.0;8.3] | 0.0 [0.0;33.3] | 0.0 [0.0;33.3] |
| Financial difficulties | 0.0 [0.0;33.3] | 0.0 [0.0;33.3] | 0.0 [0.0;66.7] | 0.0 [0.0;66.7] | 0.0 [0.0;66.7] | 0.0 [0.0;33.3] |

***** Analysis of variance or Kruskall-Wallis- and chi-squared-tests for patients with different tumor site: p<0.05

Missing for marital status (2, 4), highest graduation (3, 8) and tumor site (0, 3) for control and intervention, respectively

SD: standard deviation, Q1: 1. quartile, Q3: 3. quartile, QoL: quality of life

POS: Palliative Outcome Scale (range: 0-40, higher values = higher burden)

HADS: Hospital Anxiety and Depression Scale (range: 0-21; higher values = higher burden; abnormal=11-21)

EORTC QLQ C30: European Organization for Research and Treatment of Cancer Quality of Life Questionnaire (higher values = better status for Global health status/QoL and functional scales; higher values = higher burden for symptom scales / items)

^$^ Miscellaneous tumor sites not listed (N=75)

**POS, HADS and EORTC QLQ-C30 in proportions by tumor site, and total**

| **Outcomes in proportions^§^** | **Gastrointestinal**  **N=136** | **Respiratory system**  **N=127** | **Genitourinary**  **N=70** | **Breast**  **N=40** | **Central nervous system, N=33** | **Total**  **N=406^$^** |
| --- | --- | --- | --- | --- | --- | --- |
| **POS Score** |  |  |  |  |  |  |
| 0-10 | 84 (61.8%) | 59 (46.5%) | 36 (51.4%) | 18 (45.0%) | 23 (69.7%) | 220 (54.2%) |
| 11-20 | 40 (29.4%) | 56 (44.1%) | 27 (38.6%) | 20 (50.0%) | 10 (30.3%) | 153 (37.7%) |
| 21-30 | 10 (7.4%) | 10 (7.9%) | 7 (10.0%) | 2 (5.0%) | 0 (0.0%) | 29 (7.1%) |
| 31-40 | 2 (1.5%) | 2 (1.6%) | 0 (0.0%) | 0 (0.0%) | 0 (0.0%) | 4 (1.0%) |
| **HADS Anxiety Score** |  |  |  |  |  |  |
| Normal=0-7 | 91 (66.9%) | 69 (54.3%) | 40 (57.1%) | 28 (70.0%) | 17 (51.5%) | 245 (60.3%) |
| Mild=8-10 | 28 (20.6%) | 29 (22.8%) | 20 (28.6%) | 4 (10.0%) | 12 (36.4%) | 93 (22.9%) |
| Moderate=11-14 | 11 (8.1%) | 22 (17.3%) | 7 (10.0%) | 7 (17.5%) | 3 (9.1%) | 50 (12.3%) |
| Severe=15-21 | 6 (4.4%) | 7 (5.5%) | 3 (4.3%) | 1 (2.5%) | 1 (3.0%) | 18 (4.4%) |
| **HADS Depression Score** |  |  |  |  |  |  |
| Normal=0-7 | 97 (71.3%) | 75 (59.1%) | 44 (62.9%) | 29 (72.5%) | 20 (60.6%) | 265 (65.3%) |
| Mild=8-10 | 18 (13.2%) | 19 (15.0%) | 14 (20.0%) | 4 (10.0%) | 5 (15.2%) | 60 (14.8%) |
| Moderate=11-14 | 13 (9.6%) | 22 (17.3%) | 11 (15.7%) | 6 (15.0%) | 5 (15.2%) | 57 (14.0%) |
| Severe=15-21 | 8 (5.9%) | 11 (8.7%) | 1 (1.4%) | 1 (2.5%) | 3 (9.1%) | 24 (5.9%) |
| **EORTC QLQ C30** |  |  |  |  |  |  |
| **Global health status/QoL** |  |  |  |  |  |  |
| 0-25 | 23 (16.9%) | 29 (22.8%) | 11 (15.7%) | 4 (10.0%) | 4 (12.1%) | 71 (17.5%) |
| 26-50 | 42 (30.9%) | 52 (40.9%) | 26 (37.1%) | 15 (37.5%) | 11 (33.3%) | 146 (36.0%) |
| 51-75 | 46 (33.8%) | 36 (28.3%) | 26 (37.1%) | 15 (37.5%) | 17 (51.5%) | 140 (34.5%) |
| 76-100 | 25 (18.4%) | 10 (7.9%) | 7 (10.0%) | 6 (15.0%) | 1 (3.0%) | 49 (12.1%) |
| **Functional scales** |  |  |  |  |  |  |
| Physical functioning* |  |  |  |  |  |  |
| 0-25 | 9 (6.6%) | 22 (17.3%) | 8 (11.4%) | 2 (5.0%) | 13 (39.4%) | 54 (13.3%) |
| 26-50 | 31 (22.8%) | 33 (26.0%) | 18 (25.7%) | 10 (25.0%) | 4 (12.1%) | 96 (23.6%) |
| 51-75 | 45 (33.1%) | 40 (31.5%) | 26 (37.1%) | 14 (35.0%) | 10 (30.3%) | 135 (33.3%) |
| 76-100 | 51 (37.5%) | 32 (25.2%) | 18 (25.7%) | 14 (35.0%) | 6 (18.2%) | 121 (29.8%) |
| Role functioning |  |  |  |  |  |  |
| 0-25 | 48 (35.3%) | 52 (40.9%) | 24 (34.3%) | 10 (25.0%) | 19 (57.6%) | 153 (37.7%) |
| 26-50 | 28 (20.6%) | 32 (25.2%) | 18 (25.7%) | 15 (37.5%) | 5 (15.2%) | 98 (24.1%) |
| 51-75 | 28 (20.6%) | 21 (16.5%) | 11 (15.7%) | 9 (22.5%) | 7 (21.2%) | 76 (18.7%) |
| 76-100 | 32 (23.5%) | 22 (17.3%) | 17 (24.3%) | 6 (15.0%) | 2 (6.1%) | 79 (19.5%) |
| Emotional functioning |  |  |  |  |  |  |
| 0-25 | 21 (15.4%) | 23 (18.1%) | 11 (15.7%) | 7 (17.5%) | 5 (15.2%) | 67 (16.5%) |
| 26-50 | 29 (21.3%) | 35 (27.6%) | 22 (31.4%) | 10 (25.0%) | 13 (39.4%) | 109 (26.8%) |
| 51-75 | 44 (32.4%) | 48 (37.8%) | 21 (30.0%) | 10 (25.0%) | 11 (33.3%) | 134 (33.0%) |
| 76-100 | 42 (30.9%) | 21 (16.5%) | 16 (22.9%) | 13 (32.5%) | 4 (12.1%) | 96 (23.6%) |
| Cognitive functioning |  |  |  |  |  |  |
| 0-25 | 6 (4.4%) | 9 (7.1%) | 4 (5.7%) | 1 (2.5%) | 5 (15.2%) | 25 (6.2%) |
| 26-50 | 24 (17.6%) | 30 (23.6%) | 13 (18.6%) | 9 (22.5%) | 8 (24.2%) | 84 (20.7%) |
| 51-75 | 19 (14.0%) | 21 (16.5%) | 12 (17.1%) | 5 (12.5%) | 10 (30.3%) | 67 (16.5%) |
| 76-100 | 87 (64.0%) | 67 (52.8%) | 41 (58.6%) | 25 (62.5%) | 10 (30.3%) | 230 (56.7%) |
| Social functioning* |  |  |  |  |  |  |
| 0-25 | 32 (23.5%) | 36 (28.3%) | 16 (22.9%) | 4 (10.0%) | 16 (48.5%) | 104 (25.6%) |
| 26-50 | 33 (24.3%) | 45 (35.4%) | 25 (35.7%) | 18 (45.0%) | 9 (27.3%) | 130 (32.0%) |
| 51-75 | 28 (20.6%) | 20 (15.7%) | 11 (15.7%) | 6 (15.0%) | 4 (12.1%) | 69 (17.0%) |
| 76-100 | 43 (31.6%) | 26 (20.5%) | 18 (25.7%) | 12 (30.0%) | 4 (12.1%) | 103 (25.4%) |
| **Symptom scales** |  |  |  |  |  |  |
| Fatigue |  |  |  |  |  |  |
| 0-25 | 31 (22.8%) | 19 (15.0%) | 7 (10.0%) | 8 (20.0%) | 2 (6.1%) | 67 (16.5%) |
| 26-50 | 34 (25.0%) | 28 (22.0%) | 18 (25.7%) | 13 (32.5%) | 12 (36.4%) | 105 (25.9%) |
| 51-75 | 31 (22.8%) | 31 (24.4%) | 20 (28.6%) | 11 (27.5%) | 6 (18.2%) | 99 (24.4%) |
| 76-100 | 40 (29.4%) | 49 (38.6%) | 25 (35.7%) | 8 (20.0%) | 13 (39.4%) | 135 (33.3%) |
| Nausea and vomiting |  |  |  |  |  |  |
| 0-25 | 97 (71.3%) | 90 (70.9%) | 56 (80.0%) | 29 (72.5%) | 29 (87.9%) | 301 (74.1%) |
| 26-50 | 28 (20.6%) | 26 (20.5%) | 9 (12.9%) | 6 (15.0%) | 3 (9.1%) | 72 (17.7%) |
| 51-75 | 3 (2.2%) | 4 (3.1%) | 1 (1.4%) | 3 (7.5%) | 0 (0.0%) | 11 (2.7%) |
| 76-100 | 8 (5.9%) | 7 (5.5%) | 4 (5.7%) | 2 (5.0%) | 1 (3.0%) | 22 (5.4%) |
| Pain* |  |  |  |  |  |  |
| 0 | 70 (51.5%) | 48 (37.8%) | 20 (28.6%) | 13 (32.5%) | 22 (66.7%) | 173 (42.6%) |
| 33.3 | 33 (24.3%) | 35 (27.6%) | 22 (31.4%) | 9 (22.5%) | 6 (18.2%) | 105 (25.9%) |
| 66.6 | 11 (8.1%) | 15 (11.8%) | 16 (22.9%) | 10 (25.0%) | 3 (9.1%) | 55 (13.5%) |
| 100 | 22 (16.2%) | 29 (22.8%) | 12 (17.1%) | 8 (20.0%) | 2 (6.1%) | 73 (18.0%) |
| Dyspnea* |  |  |  |  |  |  |
| 0 | 73 (53.7%) | 39 (30.7%) | 30 (42.9%) | 14 (35.0%) | 23 (69.7%) | 179 (44.1%) |
| 33.3 | 38 (27.9%) | 32 (25.2%) | 13 (18.6%) | 15 (37.5%) | 5 (15.2%) | 103 (25.4%) |
| 66.6 | 18 (13.2%) | 29 (22.8%) | 18 (25.7%) | 9 (22.5%) | 4 (12.1%) | 78 (19.2%) |
| 100 | 7 (5.1%) | 27 (21.3%) | 9 (12.9%) | 2 (5.0%) | 1 (3.0%) | 46 (11.3%) |
| Insomnia |  |  |  |  |  |  |
| 0 | 47 (34.6%) | 33 (26.0%) | 19 (27.1%) | 15 (37.5%) | 14 (42.4%) | 128 (31.5%) |
| 33.3 | 36 (26.5%) | 28 (22.0%) | 16 (22.9%) | 12 (30.0%) | 5 (15.2%) | 97 (23.9%) |
| 66.6 | 28 (20.6%) | 30 (23.6%) | 21 (30.0%) | 8 (20.0%) | 8 (24.2%) | 95 (23.4%) |
| 100 | 25 (18.4%) | 36 (28.3%) | 14 (20.0%) | 5 (12.5%) | 6 (18.2%) | 86 (21.2%) |
| Appetite loss |  |  |  |  |  |  |
| 0 | 59 (43.4%) | 54 (42.5%) | 36 (51.4%) | 18 (45.0%) | 24 (72.7%) | 191 (47.0%) |
| 33.3 | 28 (20.6%) | 21 (16.5%) | 14 (20.0%) | 12 (30.0%) | 2 (6.1%) | 77 (19.0%) |
| 66.6 | 23 (16.9%) | 25 (19.7%) | 8 (11.4%) | 7 (17.5%) | 3 (9.1%) | 66 (16.3%) |
| 100 | 26 (19.1%) | 27 (21.3%) | 12 (17.1%) | 3 (7.5%) | 4 (12.1%) | 72 (17.7%) |
| Constipation |  |  |  |  |  |  |
| 0 | 85 (62.5%) | 77 (60.6%) | 35 (50.0%) | 19 (47.5%) | 21 (63.6%) | 237 (58.4%) |
| 33.3 | 22 (16.2%) | 19 (15.0%) | 13 (18.6%) | 10 (25.0%) | 6 (18.2%) | 70 (17.2%) |
| 66.6 | 21 (15.4%) | 24 (18.9%) | 10 (14.3%) | 4 (10.0%) | 5 (15.2%) | 64 (15.8%) |
| 100 | 8 (5.9%) | 7 (5.5%) | 12 (17.1%) | 7 (17.5%) | 1 (3.0%) | 35 (8.6%) |
| Diarrhea |  |  |  |  |  |  |
| 0 | 87 (64.0%) | 103 (81.1%) | 52 (74.3%) | 30 (75.0%) | 24 (72.7%) | 296 (72.9%) |
| 33.3 | 24 (17.6%) | 11 (8.7%) | 11 (15.7%) | 8 (20.0%) | 6 (18.2%) | 60 (14.8%) |
| 66.6 | 15 (11.0%) | 11 (8.7%) | 3 (4.3%) | 1 (2.5%) | 3 (9.1%) | 33 (8.1%) |
| 100 | 10 (7.4%) | 2 (1.6%) | 4 (5.7%) | 1 (2.5%) | 0 (0.0%) | 17 (4.2%) |
| Financial difficulties |  |  |  |  |  |  |
| 0 | 76 (55.9%) | 72 (56.7%) | 37 (52.9%) | 22 (55.0%) | 19 (57.6%) | 226 (55.7%) |
| 33.3 | 34 (25.0%) | 27 (21.3%) | 12 (17.1%) | 5 (12.5%) | 3 (9.1%) | 81 (20.0%) |
| 66.6 | 12 (8.8%) | 19 (15.0%) | 11 (15.7%) | 10 (25.0%) | 6 (18.2%) | 58 (14.3%) |
| 100 | 14 (10.3%) | 9 (7.1%) | 10 (14.3%) | 3 (7.5%) | 5 (15.2%) | 41 (10.1%) |

* Chi-squared-tests between patients with different tumor site: p<0.05

^$^ Miscellaneous tumor sites not listed (N=75)

§ POS and EORTC QLQ-C30 in four equal proportions: Global health status/QoL, functional scales and fatigue; other symptom scales of EORTC QLQ-C30: only four values: 0, 33.3, 66.6, 100; HADS according to clinical classification

POS: Palliative Outcome Scale (range: 0-40, higher values = higher burden)

HADS: Hospital Anxiety and Depression Scale (range: 0-21; higher values = higher burden)

EORTC QLQ C30: European Organization for Research and Treatment of Cancer Quality of Life Questionnaire (higher values = better status for Global health status/QoL and functional scales; higher values = higher burden for symptom scales / items)

**POS free text by tumor site, and total**

| **Most relevant problem in the past three days** | **Gastrointestinal**  **N=93** | **Respiratory system, N=93** | **Genitourinary**  **N=51** | **Breast**  **N=33** | **Central nervous system, N=24** | **Total**  **N=294** |
| --- | --- | --- | --- | --- | --- | --- |
| Physical symptoms (pain, dyspnea etc.) | 28 (30.1%) | 26 (28.0%) | 16 (31.4%) | 11 (33.3%) | 4 (16.7%) | 85 (28.9%) |
| Fear from adverse events (e.g. chemotherapy, radiation) | 9 (9.7%) | 11 (11.8%) | 4 (7.8%) | 2 (6.1%) | 2 (8.3%) | 28 (9.5%) |
| Disease and death, dealing with illness | 10 (10.8%) | 6 (6.5%) | 4 (7.8%) | 4 (12.1%) | 2 (8.3%) | 26 (8.8%) |
| Life expectancy, prognosis, chances for cure / relief | 9 (9.7%) | 6 (6.5%) | 3 (5.9%) | 2 (6.1%) | 3 (12.5%) | 23 (7.8%) |
| Financial concerns | 4 (4.3%) | 5 (5.4%) | 3 (5.9%) | 4 (12.1%) | 4 (16.7%) | 20 (6.8%) |
| No problems | 7 (7.5%) | 6 (6.5%) | 4 (7.8%) | 1 (3.0%) | 0 (0.0%) | 18 (6.1%) |
| Miscellaneous problems | 3 (3.2%) | 5 (5.4%) | 8 (15.7%) | 0 (0.0%) | 0 (0.0%) | 16 (5.4%) |
| Concerns about family and relatives | 6 (6.5%) | 4 (4.3%) | 3 (5.9%) | 1 (3.0%) | 0 (0.0%) | 14 (4.8%) |
| Home care, autonomy | 1 (1.1%) | 5 (5.4%) | 2 (3.9%) | 1 (3.0%) | 5 (20.8%) | 14 (4.8%) |
| Therapeutic decision | 2 (2.2%) | 8 (8.6%) | 1 (2.0%) | 1 (3.0%) | 0 (0.0%) | 12 (4.1%) |
| Uncertainty and concerns about future | 4 (4.3%) | 3 (3.2%) | 2 (3.9%) | 1 (3.0%) | 1 (4.2%) | 11 (3.7%) |
| Organization (e.g. transport to hospital, scheduling) | 1 (1.1%) | 5 (5.4%) | 0 (0.0%) | 2 (6.1%) | 1 (4.2%) | 9 (3.1%) |
| Psychological burden (depression, anxiety) | 3 (3.2%) | 2 (2.2%) | 0 (0.0%) | 2 (6.1%) | 1 (4.2%) | 8 (2.7%) |
| Concern about work | 5 (5.4%) | 0 (0.0%) | 1 (2.0%) | 1 (3.0%) | 1 (4.2%) | 8 (2.7%) |
| Reasons of disease | 1 (1.1%) | 1 (1.1%) | 0 (0.0%) | 0 (0.0%) | 0 (0.0%) | 2 (0.7%) |

Problems listed in descending order according to the total number and percent

Percentages refer to number in column

# Online Resource 5: Subgroup analysis: gender

**Patient characteristics by gender, and total**

| **Characteristics** | **Female N=245** | **Male N=236** | **Total N=481** |
| --- | --- | --- | --- |
| **Age in years**, mean (SD) | 61.8 (12.9) | 62.9 (11.0) | 62.4 (12.0) |
| **Marital status*:** |  |  |  |
| single | 22 (9.1%) | 17 (7.3%) | 39 (8.2%) |
| married | 152 (62.8%) | 183 (78.5%) | 335 (70.5%) |
| divorced | 29 (12.0%) | 25 (10.7%) | 54 (11.4%) |
| widowed | 39 (16.1%) | 8 (3.4%) | 47 (9.9%) |
| **Highest graduation (total years in school)*:** |  |  |  |
| general secondary school (8 years) | 99 (41.4%) | 109 (47.2%) | 208 (44.3%) |
| secondary school (10 years) | 73 (30.5%) | 41 (17.7%) | 114 (24.3%) |
| high school (12 years) | 18 (7.5%) | 27 (11.7%) | 45 (9.6%) |
| high school (13 years) | 41 (17.2%) | 49 (21.2%) | 90 (19.1%) |
| miscellaneous | 8 (3.4%) | 5 (2.2%) | 13 (2.8%) |
| **Tumor site:** |  |  |  |
| gastrointestinal | 48 (19.8%) | 88 (37.4%) | 136 (28.5%) |
| respiratory system | 51 (21.0%) | 76 (32.3%) | 127 (26.6%) |
| breast | 39 (16.0%) | 1 (0.4%) | 40 (8.4%) |
| genitourinary | 61 (25.1%) | 9 (3.8%) | 70 (14.6%) |
| central nervous system | 16 (6.58%) | 17 (7.2%) | 33 (6.9%) |
| miscellaneous | 28 (11.5%) | 44 (18.7%) | 72 (15.1%) |
|  |  |  |  |
| **POS Score***, mean (SD) | 11.7 (6.6) | 10.5 (6.1) | 11.1 (6.4) |
| **HADS Anxiety Score***, mean (SD) | 7.2 (4.3) | 6.2 (4.0) | 6.7 (4.2) |
| **HADS Depression Score**, mean (SD) | 6.8 (4.9) | 6.3 (4.5) | 6.5 (4.7) |
| **EORTC QLQ C30** |  |  |  |
| **Global health status/QoL**, mean (SD) | 50.5 (23.1) | 50.6 (24.6) | 50.6 (23.9) |
| **Functional scales**, median [Q_1_, Q_3_] |  |  |  |
| Physical functioning | 60.0 [33.3;80.0] | 66.7 [40.0;86.7] | 60.0 [33.3;83.3] |
| Role functioning | 33.3 [16.7;66.7] | 33.3 [0.0;66.7] | 33.3 [0;66.7] |
| Emotional functioning* | 58.3 [33.3;75.0] | 58.3 [41.7;83.3] | 58.3 [33.3;75.0] |
| Cognitive functioning | 83.3 [50.0;100.0] | 83.3 [66.7;100.0] | 83.3 [50.0;100.0] |
| Social functioning | 50.0 [16.7;66.7] | 50.0 [33.3;83.3] | 50.0 [33.3;77.8] |
| **Symptom scales**, median [Q_1_, Q_3_] |  |  |  |
| Fatigue* | 66.7 [44.4;77.8] | 55.6 [33.3;77.8] | 55.6 [33.3;77.8] |
| Nausea and vomiting | 0.0 [0.0;33.3] | 0.0 [0.0;16.7] | 0.0 [0.0;16.7] |
| Pain | 33.3 [0.0;66.7] | 33.3 [0.0;66.7] | 33.3 [0.0;66.7] |
| Dyspnea | 33.3 [0.0;66.7] | 33.3 [0.0;66.7] | 33.3 [0.0;66.7] |
| Insomnia | 33.3 [0.0;66.7] | 33.3 [0.0;66.7] | 33.3 [0.0;66.7] |
| Appetite loss | 33.3 [0.0;66.7] | 0.0 [0.0;66.7] | 33.3 [0.0;66.7] |
| Constipation | 0.0 [0.0;66.7] | 0.0 [0.0;33.3] | 0.0 [0.0;33.3] |
| Diarrhea | 0.0 [0.0;33.3] | 0.0 [0.0;33.3] | 0.0 [0.0;33.3] |
| Financial difficulties | 0.0 [0.0;66.7] | 0.0 [0.0;33.3] | 0.0 [0.0;33.3] |

* t-, chi-squared- or Kruskall-Wallis-tests for difference between female and male patients: p<0.05

Missing for marital status (2, 4), highest graduation (3, 8) and tumor site (0, 3) for control and intervention, respectively

SD: standard deviation, Q1: 1. quartile, Q3: 3. quartile, QoL: quality of life

POS: Palliative Outcome Scale (range: 0-40, higher values = higher burden)

HADS: Hospital Anxiety and Depression Scale (range: 0-21; higher values = higher burden; abnormal=11-21)

EORTC QLQ C30: European Organization for Research and Treatment of Cancer Quality of Life Questionnaire (higher values = better status for Global health status/QoL and functional scales; higher values = higher burden for symptom scales / items)

**POS, HADS and EORTC QLQ-C30 in proportions by sex, and total**

| **Outcomes in proportions^$^** | **Female N=245** | **Male N=236** | **Total N=481** |
| --- | --- | --- | --- |
| **POS Score** |  |  |  |
| 0-10 | 120 (49.0%) | 135 (57.2%) | 255 (53.0%) |
| 11-20 | 101 (41.2%) | 84 (35.6%) | 185 (38.5%) |
| 21-30 | 19 (7.8%) | 17 (7.2%) | 36 (7.5%) |
| 31-40 | 5 (2.0%) | 0 (0.0%) | 5 (1.0%) |
| **HADS Anxiety Score** |  |  |  |
| Normal=0-7 | 136 (55.5%) | 156 (66.1%) | 292 (60.7%) |
| Mild=8-10 | 58 (23.7%) | 46 (19.5%) | 104 (21.6%) |
| Moderate=11-14 | 37 (15.1%) | 24 (10.2%) | 61 (12.7%) |
| Severe=15-21 | 14 (5.7%) | 10 (4.2%) | 24 (5.0%) |
| **HADS Depression Score** |  |  |  |
| Normal=0-7 | 155 (63.3%) | 159 (67.4%) | 314 (65.3%) |
| Mild=8-10 | 35 (14.3%) | 34 (14.4%) | 69 (14.3%) |
| Moderate=11-14 | 34 (13.9%) | 31 (13.1%) | 65 (13.5%) |
| Severe=15-21 | 21 (8.6%) | 12 (5.1%) | 33 (6.9%) |
| **EORTC QLQ C30** |  |  |  |
| **Global health status/QoL** |  |  |  |
| 0-25 | 42 (17.1%) | 47 (19.9%) | 89 (18.5%) |
| 26-50 | 97 (39.6%) | 76 (32.2%) | 175 (36.0%) |
| 51-75 | 83 (33.9%) | 75 (31.8%) | 158 (32.8%) |
| 76-100 | 23 (9.4%) | 38 (16.1%) | 61 (12.7%) |
| **Functional scales** |  |  |  |
| Physical functioning |  |  |  |
| 0-25 | 31 (12.7%) | 33 (14.0%) | 64 (13.3%) |
| 26-50 | 62 (25.3%) | 47 (19.9%) | 109 (22.7%) |
| 51-75 | 88 (35.9%) | 70 (29.7%) | 158 (32.8%) |
| 76-100 | 64 (26.1%) | 86 (36.4%) | 150 (31.2%) |
| Role functioning |  |  |  |
| 0-25 | 88 (35.9%) | 92 (39.0%) | 180 (37.4%) |
| 26-50 | 66 (26.9%) | 53 (22.5%) | 119 (24.7%) |
| 51-75 | 42 (17.1%) | 43 (18.2%) | 85 (17.7%) |
| 76-100 | 49 (20.0%) | 48 (20.3%) | 97 (20.2%) |
| Emotional functioning |  |  |  |
| 0-25 | 49 (20.0%) | 34 (14.4%) | 83 (17.3%) |
| 26-50 | 67 (27.3%) | 57 (24.2%) | 124 (25.8%) |
| 51-75 | 81 (33.1%) | 78 (33.1%) | 159 (33.1%) |
| 76-100 | 48 (19.6%) | 67 (28.4%) | 115 (23.9%) |
| Cognitive functioning |  |  |  |
| 0-25 | 19 (7.8%) | 10 (4.2%) | 29 (6.0%) |
| 26-50 | 56 (22.9%) | 43 (18.2%) | 99 (20.6%) |
| 51-75 | 36 (14.7%) | 46 (19.5%) | 82 (17.0%) |
| 76-100 | 134 (54.7%) | 137 (58.1%) | 271 (56.3%) |
| Social functioning |  |  |  |
| 0-25 | 70 (28.6%) | 56 (23.7%) | 126 (26.2%) |
| 26-50 | 76 (31.0%) | 71 (30.1%) | 147 (30.6%) |
| 51-75 | 40 (16.3%) | 41 (17.4%) | 81 (16.8%) |
| 76-100 | 59 (24.1%) | 68 (28.8%) | 127 (26.4%) |
| **Symptom scales** |  |  |  |
| Fatigue |  |  |  |
| 0-25 | 32 (13.1%) | 53 (22.5%) | 85 (17.7%) |
| 26-50 | 63 (25.7%) | 56 (23.7%) | 119 (24.7%) |
| 51-75 | 61 (24.9%) | 54 (22.9%) | 115 (23.9%) |
| 76-100 | 89 (36.3%) | 73 (30.9%) | 162 (33.7%) |
| Nausea and vomiting |  |  |  |
| 0-25 | 175 (71.4%) | 186 (78.8%) | 361 (75.1%) |
| 26-50 | 46 (18.8%) | 34 (14.4%) | 80 (16.6%) |
| 51-75 | 7 (2.9%) | 4 (1.7%) | 11 (2.3%) |
| 76-100 | 17 (6.9%) | 12 (5.1%) | 29 (6.0%) |
| Pain |  |  |  |
| 0 | 97 (39.6%) | 105 (44.5%) | 202 (42.0%) |
| 33.3 | 62 (25.3%) | 62 (26.3%) | 124 (25.8%) |
| 66.6 | 37 (15.1%) | 30 (12.7%) | 67 (13.9%) |
| 100 | 49 (20.0%) | 39 (16.5%) | 88 (18.3%) |
| Dyspnea |  |  |  |
| 0 | 104 (42.4%) | 110 (46.6%) | 214 (44.5%) |
| 33.3 | 64 (26.1%) | 56 (23.7%) | 120 (24.9%) |
| 66.6 | 44 (18.0%) | 44 (18.6%) | 88 (18.3%) |
| 100 | 33 (13.5%) | 26 (11.0%) | 59 (12.3%) |
| Insomnia |  |  |  |
| 0 | 87 (35.5%) | 72 (30.5%) | 159 (33.1%) |
| 33.3 | 53 (21.6%) | 56 (23.7%) | 109 (22.7%) |
| 66.6 | 53 (21.6%) | 59 (25.0%) | 112 (23.3%) |
| 100 | 52 (21.2%) | 49 (20.8%) | 101 (21.0%) |
| Appetite loss |  |  |  |
| 0 | 113 (46.1%) | 123 (52.1%) | 236 (49.1%) |
| 33.3 | 51 (20.8%) | 35 (14.8%) | 86 (17.9%) |
| 66.6 | 33 (13.5%) | 43 (18.2%) | 76 (15.8%) |
| 100 | 48 (19.6%) | 35 (14.8%) | 83 (17.3%) |
| Constipation* |  |  |  |
| 0 | 137 (55.9%) | 151 (64.0%) | 288 (59.9%) |
| 33.3 | 46 (18.8%) | 29 (12.3%) | 75 (15.6%) |
| 66.6 | 33 (13.5%) | 43 (18.2%) | 76 (15.8%) |
| 100 | 29 (11.8%) | 13 (5.5%) | 42 (8.7%) |
| Diarrhea |  |  |  |
| 0 | 178 (72.7%) | 173 (73.3%) | 351 (73.0%) |
| 33.3 | 35 (14.3%) | 32 (13.6%) | 67 (13.9%) |
| 66.6 | 22 (9.0%) | 22 (9.3%) | 44 (9.1%) |
| 100 | 10 (4.1%) | 9 (3.8%) | 19 (4.0%) |
| Financial difficulties |  |  |  |
| 0 | 139 (56.7%) | 135 (57.2%) | 274 (57.0%) |
| 33.3 | 42 (17.1%) | 48 (20.3%) | 90 (18.7%) |
| 66.6 | 38 (15.5%) | 34 (14.4%) | 72 (15.0%) |
| 100 | 26 (10.6%) | 19 (8.1%) | 45 (9.4%) |

***** Chi-squared-tests for female and male patients: p<0.05

^$^ POS and EORTC QLQ-C30 in four equal proportions: Global health status/QoL, functional scales and fatigue; other symptom scales of EORTC QLQ-C30: only four values: 0, 33.3, 66.6, 100; HADS according to clinical classification

POS: Palliative Outcome Scale (range: 0-40, higher values = higher burden)

HADS: Hospital Anxiety and Depression Scale (range: 0-21; higher values = higher burden)

EORTC QLQ C30: European Organization for Research and Treatment of Cancer Quality of Life Questionnaire (higher values = better status for Global health status/QoL and functional scales; higher values = higher burden for symptom scales / items)

**POS free text by sex, and total**

| **Most relevant problem in the past three days** | **Female**  **N=187** | **Male**  **N=160** | **Total**  **N=347** |
| --- | --- | --- | --- |
| Physical symptoms (pain, dyspnea etc.) | 50 (26.7%) | 43 (26.9%) | 93 (26.8%) |
| Fear from adverse events (e.g. chemotherapy, radiation) | 22 (11.8%) | 10 (6.3%) | 32 (9.2%) |
| Disease and death, dealing with illness | 18 (9.6%) | 12 (7.5%) | 30 (8.7%) |
| Life expectancy, prognosis, chances for cure / relief | 12 (6.4%) | 16 (10.0%) | 28 (8.1%) |
| Financial concerns | 12 (6.4%) | 11 (6.9%) | 23 (6.6%) |
| No problems | 11 (5.9%) | 10 (6.3%) | 21 (6.1%) |
| Miscellaneous problems | 9 (4.8%) | 11 (6.9%) | 20 (5.8%) |
| Therapeutic decision | 9 (4.8%) | 10 (6.3%) | 19 (5.5%) |
| Concerns about family and relatives | 11 (5.9%) | 6 (3.8%) | 17 (4.9%) |
| Home care, autonomy | 7 (3.7%) | 8 (5.0%) | 15 (4.3%) |
| Uncertainty and concerns about future | 6 (3.2%) | 8 (5.0%) | 14 (4.0%) |
| Psychological burden (depression, anxiety) | 7 (3.7%) | 5 (3.1%) | 12 (3.5%) |
| Organization (e.g. transport to hospital, scheduling) | 7 (3.7%) | 4 (2.5%) | 11 (3.2%) |
| Concern about work | 6 (3.2%) | 3 (1.9%) | 9 (2.6%) |
| Reasons of disease | 0 (0.0%) | 3 (1.9%) | 3 (0.9%) |

Problems listed in descending order according to the total number and percent

Percentages refer to number in column

# Online Resource 6: Subgroup analysis: age

**Patient characteristics by median age, and total**

| **Characteristics** | **<65 N=273** | **>=65 N=208** | **Total N=481** |
| --- | --- | --- | --- |
| **Age in years***, mean (SD) | 54.0 (8.1) | 73.3 (6.0) | 62.4 (12.0) |
| **Sex:** |  |  |  |
| female | 142 (52.0%) | 103 (49.5%) | 245 (50.9%) |
| male | 131 (48.0%) | 105 (50.5%) | 236 (49.1%) |
| **Marital status:** |  |  |  |
| single | 32 (11.8%) | 7 (3.4%) | 39 (8.2%) |
| married | 197 (72.7%) | 138 (67.6%) | 335 (70.5%) |
| divorced | 35 (12.9%) | 19 (9.3%) | 54 (11.4%) |
| widowed | 7 (2.6%) | 40 (19.6%) | 47 (9.9%) |
| **Highest graduation* (total years in school):** |  |  |  |
| general secondary school (8 years) | 98 (36.4%) | 110 (54.7%) | 208 (44.3%) |
| secondary school (10 years) | 71 (26.4%) | 43 (21.4%) | 114 (24.3%) |
| high school (12 years) | 25 (9.3%) | 20 (10.0%) | 45 (9.6%) |
| high school (13 years) | 67 (24.9%) | 23 (11.4%) | 90 (19.1%) |
| miscellaneous | 8 (2.97%) | 5 (2.5%) | 13 (2.8%) |
| **Tumor site:** |  |  |  |
| gastrointestinal | 73 (26.9%) | 63 (30.4%) | 136 (28.5%) |
| respiratory system | 67 (24.7%) | 60 (29.0%) | 127 (26.6%) |
| breast | 26 (9.6%) | 14 (6.8%) | 40 (8.4%) |
| genitourinary | 38 (14.0%) | 32 (15.5%) | 70 (14.6%) |
| central nervous system | 24 (8.9%) | 9 (4.4%) | 33 (6.9%) |
| miscellaneous | 43 (15.9%) | 29 (14.0%) | 72 (15.1%) |
|  |  |  |  |
| **POS Score**, mean (SD) | 10.9 (6.4) | 11.3 (6.3) | 11.1 (6.4) |
| **HADS Anxiety Score**, mean (SD) | 6.8 (4.0) | 6.6 (4.4) | 6.7 (4.2) |
| **HADS Depression Score**, mean (SD) | 6.4 (4.6) | 6.8 (4.8) | 6.5 (4.7) |
| **EORTC QLQ C30** |  |  |  |
| **Global health status/QoL**, mean (SD) | 51.5 (23.5) | 49.4 (24.3) | 50.6 (23.9) |
| **Functional scales**, median [Q_1_, Q_3_] |  |  |  |
| Physical functioning* | 66.7 [41.7;86.7] | 53.3 [33.3;80.0] | 60.0 [33.3;83.3] |
| Role functioning | 33.3 [0.0;66.7] | 33.3 [0.0;83.3] | 33.3 [0;66.7] |
| Emotional functioning | 58.3 [33.3;75.0] | 58.3 [39.6;79.2] | 58.3 [33.3;75.0] |
| Cognitive functioning | 83.3 [50.0;100.0] | 83.3 [50.0;100.0] | 83.3 [50.0;100.0] |
| Social functioning | 50.0 [16.7;66.7] | 50.0 [16.7;83.3] | 50.0 [33.3;77.8] |
| **Symptom scales**, median [Q_1_, Q_3_] |  |  |  |
| Fatigue | 55.6 [33.3;77.8] | 66.7 [33.3;88.9] | 55.6 [33.3;77.8] |
| Nausea and vomiting | 0.0 [0.0;33.3] | 0.0 [0.0;16.7] | 0.0 [0.0;16.7] |
| Pain | 33.3 [0.0;66.7] | 33.3 [0.0;66.7] | 33.3 [0.0;66.7] |
| Dyspnea | 33.3 [0.0;66.7] | 33.3 [0.0;66.7] | 33.3 [0.0;66.7] |
| Insomnia | 33.3 [0.0;66.7] | 33.3 [0.0;66.7] | 33.3 [0.0;66.7] |
| Appetite loss | 0.0 [0.0;66.7] | 33.3 [0.0;66.7] | 33.3 [0.0;66.7] |
| Constipation | 0.0 [0.0;33.3] | 0.0 [0.0;66.7] | 0.0 [0.0;33.3] |
| Diarrhea | 0.0 [0.0;33.3] | 0.0 [0.0;8.3] | 0.0 [0.0;33.3] |
| Financial difficulties* | 33.3 [0.0;66.7] | 0.0 [0.0;33.3] | 0.0 [0.0;33.3] |

***** t-, chi-squared- or Kruskall-Wallis-tests for difference between patients <63 and ≥63 years (i.e., median age): p<0.05

Missing for marital status (2, 4), highest graduation (3, 8) and tumor site (0, 3) for control and intervention, respectively

SD: standard deviation, Q1: 1. quartile, Q3: 3. quartile, QoL: quality of life

POS: Palliative Outcome Scale (range: 0-40, higher values = higher burden)

HADS: Hospital Anxiety and Depression Scale (range: 0-21; higher values = higher burden; abnormal=11-21)

EORTC QLQ C30: European Organization for Research and Treatment of Cancer Quality of Life Questionnaire (higher values = better status for Global health status/QoL and functional scales; higher values = higher burden for symptom scales/items)**POS, HADS and EORTC QLQ-C30 in proportions by median age, and total**

| **Outcomes in proportions^$^** | **<65 N=273** | **>=65 N=208** | **Total N=481** |
| --- | --- | --- | --- |
| **POS Score** |  |  |  |
| 0-10 | 153 (56.0%) | 102 (49.0%) | 255 (53.0%) |
| 11-20 | 97 (35.5%) | 88 (42.3%) | 185 (38.5%) |
| 21-30 | 20 (7.3%) | 16 (7.7%) | 36 (7.5%) |
| 31-40 | 3 (1.1%) | 2 (1.0%) | 5 (1.0%) |
| **HADS Anxiety Score** |  |  |  |
| Normal=0-7 | 160 (58.6%) | 132 (63.5%) | 292 (60.7%) |
| Mild=8-10 | 69 (25.3%) | 35 (16.8%) | 104 (21.6%) |
| Moderate=11-14 | 31 (11.4%) | 30 (14.4%) | 61 (12.7%) |
| Severe=15-21 | 13 (4.8%) | 11 (5.3%) | 24 (5.0%) |
| **HADS Depression Score** |  |  |  |
| Normal=0-7 | 182 (66.7%) | 132 (63.5%) | 314 (65.3%) |
| Mild=8-10 | 37 (13.6%) | 32 (15.4%) | 69 (14.3%) |
| Moderate=11-14 | 37 (13.6%) | 28 (13.5%) | 65 (13.5%) |
| Severe=15-21 | 17 (6.2%) | 16 (7.7%) | 33 (6.9%) |
| **EORTC QLQ C30** |  |  |  |
| **Global health status/QoL** |  |  |  |
| 0-25 | 45 (16.5%) | 44 (21.2%) | 89 (18.5%) |
| 26-50 | 101 (37.0%) | 72 (34.6%) | 175 (36.0%) |
| 51-75 | 93 (34.1%) | 65 (31.2%) | 158 (32.8%) |
| 76-100 | 34 (12.5%) | 27 (13.0%) | 61 (12.7%) |
| **Functional scales** |  |  |  |
| Physical functioning* |  |  |  |
| 0-25 | 26 (9.5%) | 38 (18.3%) | 64 (13.3%) |
| 26-50 | 57 (20.9%) | 52 (25.0%) | 109 (22.7%) |
| 51-75 | 101 (37.0%) | 57 (27.4%) | 158 (32.8%) |
| 76-100 | 89 (32.6%) | 61 (29.3%) | 150 (31.2%) |
| Role functioning* |  |  |  |
| 0-25 | 103 (37.7%) | 77 (37.0%) | 180 (37.4%) |
| 26-50 | 71 (26.0%) | 48 (23.1%) | 119 (24.7%) |
| 51-75 | 56 (20.5%) | 29 (13.9%) | 85 (17.7%) |
| 76-100 | 43 (15.8%) | 54 (26.0%) | 97 (20.2%) |
| Emotional functioning |  |  |  |
| 0-25 | 50 (18.3%) | 33 (15.9%) | 83 (17.3%) |
| 26-50 | 73 (26.7%) | 51 (24.5%) | 124 (25.8%) |
| 51-75 | 88 (32.2%) | 71 (34.1%) | 159 (33.1%) |
| 76-100 | 62 (22.7%) | 53 (25.5%) | 115 (23.9%) |
| Cognitive functioning |  |  |  |
| 0-25 | 17 (6.2%) | 12 (5.8%) | 29 (6.0%) |
| 26-50 | 55 (20.1%) | 44 (21.2%) | 99 (20.6%) |
| 51-75 | 52 (19.0%) | 30 (14.4%) | 82 (17.0%) |
| 76-100 | 149 (54.6%) | 122 (58.7%) | 271 (56.3%) |
| Social functioning |  |  |  |
| 0-25 | 71 (26.0%) | 55 (26.4%) | 126 (26.2%) |
| 26-50 | 90 (33.0%) | 57 (27.4%) | 147 (30.6%) |
| 51-75 | 45 (16.5%) | 36 (17.3%) | 81 (16.8%) |
| 76-100 | 67 (24.5%) | 60 (28.8%) | 127 (26.4%) |
| **Symptom scales** |  |  |  |
| Fatigue |  |  |  |
| 0-25 | 42 (15.4%) | 43 (20.7%) | 85 (17.7%) |
| 26-50 | 80 (29.3%) | 39 (18.8%) | 119 (24.7%) |
| 51-75 | 68 (24.9%) | 47 (22.6%) | 115 (23.9%) |
| 76-100 | 83 (30.4%) | 79 (38.0%) | 162 (33.7%) |
| Nausea and vomiting |  |  |  |
| 0-25 | 201 (73.6%) | 160 (76.9%) | 361 (75.1%) |
| 26-50 | 51 (18.7%) | 29 (13.9%) | 80 (16.6%) |
| 51-75 | 5 (1.8%) | 6 (2.9%) | 11 (2.3%) |
| 76-100 | 16 (5.9%) | 13 (6.2%) | 29 (6.0%) |
| Pain |  |  |  |
| 0 | 113 (41.4%) | 89 (42.8%) | 202 (42.0%) |
| 33.3 | 72 (26.4%) | 52 (25.0%) | 124 (25.8%) |
| 66.6 | 38 (13.9%) | 29 (13.9%) | 67 (13.9%) |
| 100 | 50 (18.3%) | 38 (18.3%) | 88 (18.3%) |
| Dyspnea |  |  |  |
| 0 | 113 (41.4%) | 101 (48.6%) | 214 (44.5%) |
| 33.3 | 76 (27.8%) | 44 (21.2%) | 120 (24.9%) |
| 66.6 | 52 (19.0%) | 36 (17.3%) | 88 (18.3%) |
| 100 | 32 (11.7%) | 27 (13.0%) | 59 (12.3%) |
| Insomnia |  |  |  |
| 0 | 85 (31.1%) | 74 (35.6%) | 159 (33.1%) |
| 33.3 | 67 (24.5%) | 42 (20.2%) | 109 (22.7%) |
| 66.6 | 63 (23.1%) | 49 (23.6%) | 112 (23.3%) |
| 100 | 58 (21.2%) | 43 (20.7%) | 101 (21.0%) |
| Appetite loss |  |  |  |
| 0 | 143 (52.4%) | 93 (44.7%) | 236 (49.1%) |
| 33.3 | 49 (17.9%) | 37 (17.8%) | 86 (17.9%) |
| 66.6 | 44 (16.1%) | 32 (15.4%) | 76 (15.8%) |
| 100 | 37 (13.6%) | 46 (22.1%) | 83 (17.3%) |
| Constipation |  |  |  |
| 0 | 169 (61.9%) | 119 (57.2%) | 288 (59.9%) |
| 33.3 | 45 (16.5%) | 30 (14.4%) | 75 (15.6%) |
| 66.6 | 42 (15.4%) | 34 (16.3%) | 76 (15.8%) |
| 100 | 17 (6.2%) | 25 (12.0%) | 42 (8.7%) |
| Diarrhea |  |  |  |
| 0 | 195 (71.4%) | 156 (75.0%) | 351 (73.0%) |
| 33.3 | 43 (15.8%) | 24 (11.5%) | 67 (13.9%) |
| 66.6 | 25 (9.2%) | 19 (9.1%) | 44 (9.1%) |
| 100 | 10 (3.7%) | 9 (4.3%) | 19 (4.0%) |
| Financial difficulties* |  |  |  |
| 0 | 131 (48.0%) | 143 (68.8%) | 274 (57.0%) |
| 33.3 | 55 (20.1%) | 35 (16.8%) | 90 (18.7%) |
| 66.6 | 52 (19.0%) | 20 (9.6%) | 72 (15.0%) |
| 100 | 35 (12.8%) | 10 (4.8%) | 45 (9.4%) |

***** Chi-squared-tests for patients <63 and ≥63 years (i.e., median age): p<0.05

^$^ POS and EORTC QLQ-C30 in four equal proportions: Global health status/QoL, functional scales and fatigue; other symptom scales of EORTC QLQ-C30: only four values: 0, 33.3, 66.6, 100; HADS according to clinical classification

POS: Palliative Outcome Scale (range: 0-40, higher values = higher burden)

HADS: Hospital Anxiety and Depression Scale (range: 0-21; higher values = higher burden)

EORTC QLQ C30: European Organization for Research and Treatment of Cancer Quality of Life Questionnaire (higher values = better status for Global health status/QoL and functional scales; higher values = higher burden for symptom scales / items)

**POS free text by median age, and total**

| **Most relevant problem in the past three days** | **<65**  **N=197** | **>=65**  **N=150** | **Total**  **N=347** |
| --- | --- | --- | --- |
| Physical symptoms (pain, dyspnea etc.) | 53 (26.9%) | 40 (26.7%) | 93 (26.8%) |
| Fear from adverse events (e.g. chemotherapy, radiation) | 18 (9.1%) | 14 (9.3%) | 32 (9.2%) |
| Disease and death, dealing with illness | 16 (8.1%) | 14 (9.3%) | 30 (8.6%) |
| Life expectancy, prognosis, chances for cure / relief | 13 (6.6%) | 15 (10.0%) | 28 (8.1%) |
| Financial concerns | 18 (9.1%) | 5 (3.3%) | 23 (6.6%) |
| No problems | 10 (5.1%) | 11 (7.3%) | 21 (6.1%) |
| Miscellaneous problems | 10 (5.1%) | 10 (6.7%) | 20 (5.8%) |
| Therapeutic decision | 10 (5.1%) | 9 (6.0%) | 19 (5.5%) |
| Concerns about family and relatives | 3 (1.5%) | 14 (9.3%) | 17 (4.9%) |
| Home care, autonomy | 7 (3.6%) | 8 (5.3%) | 15 (4.3%) |
| Uncertainty and concerns about future | 10 (5.1%) | 4 (2.7%) | 14 (4.0%) |
| Psychological burden (depression, anxiety) | 10 (5.1%) | 2 (1.3%) | 12 (3.5%) |
| Organization (e.g. transport to hospital, scheduling) | 9 (4.6%) | 2 (1.3%) | 11 (3.2%) |
| Concern about work | 8 (4.1%) | 1 (0.7%) | 9 (2.6%) |
| Reasons of disease | 2 (1.0%) | 1 (0.7%) | 3 (0.9%) |

Problems listed in descending order according to the total number and percent

Percentages refer to number in column

# Online Resource 7: Subgroup analysis: marital status

**Patient characteristics by marital status, and total**

| **Characteristics** | **Single**  **N=39** | **Married**  **N=335** | **Divorced**  **N=54** | **Widowed**  **N=47** | **Total**  **N=475** |
| --- | --- | --- | --- | --- | --- |
| **Age in years***, mean (SD) | 54.1 (12.1) | 61.9 (11.3) | 60.4 (10.7) | 74.1 (10.0) | 62.3 (12.0) |
| **Sex*:** |  |  |  |  |  |
| female | 22 (56.4%) | 152 (45.4%) | 29 (53.7%) | 39 (83.0%) | 242 (50.9%) |
| male | 17 (43.6%) | 183 (54.6%) | 25 (46.3%) | 8 (17.0%) | 233 (49.1%) |
| **Highest graduation (total years in school):** |  |  |  |  |  |
| general secondary school (8 years) | 12 (30.8%) | 150 (45.6%) | 19 (37.3%) | 26 (55.3%) | 207 (44.4%) |
| secondary school (10 years) | 11 (28.2%) | 71 (21.6%) | 16 (31.4%) | 14 (29.8%) | 112 (24.0%) |
| high school (12 years) | 5 (12.8%) | 35 (10.6%) | 3 (5.9%) | 2 (4.3%) | 45 (9.7%) |
| high school (13 years) | 10 (25.6%) | 64 (19.5%) | 11 (21.6%) | 4 (8.5%) | 89 (19.1%) |
| miscellaneous | 1 (2.6%) | 9 (2.7%) | 2 (3.9%) | 1 (2.1%) | 13 (2.8%) |
| **Tumor site:** |  |  |  |  |  |
| gastrointestinal | 11 (28.9%) | 97 (29.1%) | 13 (24.1%) | 12 (25.5%) | 133 (28.2%) |
| respiratory system | 9 (23.7%) | 94 (28.2%) | 12 (22.2%) | 12 (25.5%) | 127 (26.9%) |
| breast | 5 (13.2%) | 26 (7.8%) | 3 (5.6%) | 5 (10.6%) | 39 (8.3%) |
| genitourinary | 5 (13.2%) | 41 (12.3%) | 13 (24.1%) | 11 (23.4%) | 70 (14.8%) |
| central nervous system | 1 (2.6%) | 26 (7.8%) | 4 (7.4%) | 1 (2.1%) | 32 (6.8%) |
| miscellaneous | 7 (18.4%) | 49 (14.7%) | 9 (16.7%) | 6 (12.8%) | 71 (15.0%) |
|  |  |  |  |  |  |
| **POS Score**, mean (SD) | 11.2 (6.0) | 10.8 (6.4) | 12.3 (6.3) | 12.0 (6.7) | 11.1 (6.4) |
| **HADS Anxiety Score**, mean (SD) | 6.8 (3.3) | 6.8 (4.3) | 6.6 (4.0) | 6.4 (4.4) | 6.7 (4.2) |
| **HADS Depression Score**, mean (SD) | 6.7 (5.1) | 6.6 (4.7) | 6.1 (4.3) | 7.0 (5.2) | 6.6 (4.7) |
| **EORTC QLQ C30** |  |  |  |  |  |
| **Global health status/QoL**, mean (SD) | 50.9 (20.7) | 50.7 (24.5) | 50.2 (22.6) | 50.0 (24.1) | 50.6 (23.9) |
| **Functional scales**, median [Q_1_, Q_3_] |  |  |  |  |  |
| Physical functioning | 73.3 [53.3;85.0] | 60.0 [33.3;86.7] | 60.0 [42.9;85.0] | 53.3 [26.7;73.3] | 60.0 [36.7;81.7] |
| Role functioning | 33.3 [16.7;66.7] | 33.3 [0.0;66.7] | 50.0 [16.7;66.7] | 33.3 [0.0;66.7] | 33.3 [0.0;66.7] |
| Emotional functioning | 58.3 [37.5;83.3] | 58.3 [33.3;75.0] | 66.7 [41.7;87.5] | 58.3 [33.3;83.3] | 58.3 [33.3;75.0] |
| Cognitive functioning | 66.7 [50.0;91.7] | 83.3 [58.3;100.0] | 66.7 [50.0;100.0] | 83.3 [50.0;100.0] | 83.3 [50.0;100.0] |
| Social functioning | 66.7 [16.7;83.3] | 50.0 [16.7;75.0] | 50.0 [33.3;83.3] | 50.0 [16.7;66.7] | 50.0 [16.7;83.3] |
| **Symptom scales**, median [Q_1_, Q_3_] |  |  |  |  |  |
| Fatigue | 55.6 [33.3;77.8] | 55.6 [33.3;77.8] | 66.7 [33.3;77.8] | 66.7 [44.4;83.3] | 55.6 [33.3;77.8] |
| Nausea and vomiting | 0.0 [0.0;33.3] | 0.0 [0.0;16.7] | 0.0 [0.0;33.3] | 0.0 [0.0;8.3] | 0.0 [0.0;16.7] |
| Pain | 33.3 [0.0;83.3] | 33.3 [0.0;66.7] | 33.3 [16.7;66.7] | 33.3 [0.0;66.7] | 33.3 [0.0;66.7] |
| Dyspnea | 33.3 [0.0;33.3] | 33.3 [0.0;66.7] | 33.3 [0.0;66.7] | 33.3 [0.0;66.7] | 33.3 [0.0;66.7] |
| Insomnia | 33.3 [0.0;66.7] | 33.3 [0.0;66.7] | 66.7 [33.3;66.7] | 33.3 [0.0;66.7] | 33.3 [0.0;66.7] |
| Appetite loss | 33.3 [0.0;66.7] | 0.0 [0.0;66.7] | 33.3 [0.0;66.7] | 33.3 [0.0;66.7] | 33.3 [0.0;66.7] |
| Constipation | 0.0 [0.0;66.7] | 0.0 [0.0;33.3] | 0.0 [0.0;66.7] | 0.0 [0.0;66.7] | 0.0 [0.0;33.3] |
| Diarrhea | 0.0 [0.0;33.3] | 0.0 [0.0;33.3] | 0.0 [0.0;33.3] | 0.0 [0.0;0.0] | 0.0 [0.0;33.3] |
| Financial difficulties* | 33.3 [0.0;66.7] | 0.0 [0.0;33.3] | 33.3 [0.0;66.7] | 0.0 [0.0;33.3] | 0.0 [0.0;33.3] |

***** Analysis of variance or Kruskall-Wallis- or chi-squared-tests for patients with different marital status: p<0.05

Missing for marital status (2, 4), highest graduation (3, 8) and tumor site (0, 3) for control and intervention, respectively

SD: standard deviation, Q1: 1. quartile, Q3: 3. quartile, QoL: quality of life

POS: Palliative Outcome Scale (range: 0-40, higher values = higher burden)

HADS: Hospital Anxiety and Depression Scale (range: 0-21; higher values = higher burden; abnormal=11-21)

EORTC QLQ C30: European Organization for Research and Treatment of Cancer Quality of Life Questionnaire (higher values = better status for Global health status/QoL and functional scales; higher values = higher burden for symptom scales / items)

**POS, HADS and EORTC QLQ-C30 in proportions by marital status, and total**

| **Outcomes in proportions^$^** | **Single**  **N=39** | **Married**  **N=335** | **Divorced**  **N=54** | **Widowed**  **N=47** | **Total**  **N=475** |
| --- | --- | --- | --- | --- | --- |
| **POS Score** |  |  |  |  |  |
| 0-10 | 17 (43.6%) | 192 (57.3%) | 23 (42.6%) | 19 (40.4%) | 251 (52.8%) |
| 11-20 | 20 (51.3%) | 115 (34.3%) | 26 (48.1%) | 23 (48.9%) | 184 (38.7%) |
| 21-30 | 2 (5.1%) | 25 (7.5%) | 4 (7.4%) | 4 (8.5%) | 35 (7.4%) |
| 31-40 | 0 (0.0%) | 3 (0.9%) | 1 (1.9%) | 1 (2.1%) | 5 (1.1%) |
| **HADS Anxiety Score** |  |  |  |  |  |
| Normal=0-7 | 21 (53.8%) | 203 (60.6%) | 32 (59.3%) | 31 (66.0%) | 287 (60.4%) |
| Mild=8-10 | 13 (33.3%) | 69 (20.6%) | 14 (25.9%) | 7 (14.9%) | 103 (21.7%) |
| Moderate=11-14 | 5 (12.8%) | 42 (12.5%) | 7 (13.0%) | 7 (14.9%) | 61 (12.8%) |
| Severe=15-21 | 0 (0.0%) | 21 (6.3%) | 1 (1.9%) | 2 (4.3%) | 24 (5.1%) |
| **HADS Depression Score** |  |  |  |  |  |
| Normal=0-7 | 25 (64.1%) | 219 (65.4%) | 36 (66.7%) | 29 (61.7%) | 309 (65.1%) |
| Mild=8-10 | 4 (10.3%) | 46 (13.7%) | 11 (20.4%) | 8 (17.0%) | 69 (14.5%) |
| Moderate=11-14 | 8 (20.5%) | 46 (13.7%) | 5 (9.3%) | 5 (10.6%) | 64 (13.5%) |
| Severe=15-21 | 2 (5.1%) | 24 (7.2%) | 2 (3.7%) | 5 (10.6%) | 33 (6.9%) |
| **EORTC QLQ C30** |  |  |  |  |  |
| **Global health status/QoL** |  |  |  |  |  |
| 0-25 | 7 (17.9%) | 63 (18.8%) | 8 (14.8%) | 10 (21.3%) | 88 (18.5%) |
| 26-50 | 15 (38.5%) | 118 (35.2%) | 21 (38.9%) | 17 (36.2%) | 171 (36.0%) |
| 51-75 | 13 (33.3%) | 109 (32.5%) | 20 (37.0%) | 14 (29.8%) | 156 (32.8%) |
| 76-100 | 4 (10.3%) | 45 (13.4%) | 5 (9.3%) | 6 (12.8%) | 60 (12.6%) |
| **Functional scales** |  |  |  |  |  |
| Physical functioning |  |  |  |  |  |
| 0-25 | 3 (7.7%) | 45 (13.4%) | 5 (9.3%) | 10 (21.3%) | 63 (13.3%) |
| 26-50 | 6 (15.4%) | 79 (23.6%) | 11 (20.4%) | 12 (25.5%) | 108 (22.7%) |
| 51-75 | 16 (41.0%) | 106 (31.6%) | 20 (37.0%) | 14 (29.8%) | 156 (32.8%) |
| 76-100 | 14 (35.9%) | 105 (31.3%) | 18 (33.3%) | 11 (23.4%) | 148 (31.2%) |
| Role functioning |  |  |  |  |  |
| 0-25 | 14 (35.9%) | 125 (37.3%) | 17 (31.5%) | 23 (48.9%) | 179 (37.7%) |
| 26-50 | 14 (35.9%) | 82 (24.5%) | 11 (20.4%) | 9 (19.1%) | 116 (24.4%) |
| 51-75 | 5 (12.8%) | 57 (17.0%) | 15 (27.8%) | 8 (17.0%) | 85 (17.9%) |
| 76-100 | 6 (15.4%) | 71 (21.2%) | 11 (20.4%) | 7 (14.9%) | 95 (20.0%) |
| Emotional functioning |  |  |  |  |  |
| 0-25 | 6 (15.4%) | 63 (18.8%) | 5 (9.3%) | 9 (19.1%) | 83 (17.5%) |
| 26-50 | 8 (20.5%) | 90 (26.9%) | 15 (27.8%) | 10 (21.3%) | 123 (25.9%) |
| 51-75 | 13 (33.3%) | 116 (34.6%) | 14 (25.9%) | 15 (31.9%) | 158 (33.3%) |
| 76-100 | 12 (30.8%) | 66 (19.7%) | 20 (37.0%) | 13 (27.7%) | 111 (23.4%) |
| Cognitive functioning* |  |  |  |  |  |
| 0-25 | 4 (10.3%) | 13 (3.9%) | 5 (9.3%) | 7 (14.9%) | 29 (6.1%) |
| 26-50 | 7 (17.9%) | 71 (21.2%) | 10 (18.5%) | 9 (19.1%) | 97 (20.4%) |
| 51-75 | 9 (23.1%) | 53 (15.8%) | 14 (25.9%) | 6 (12.8%) | 82 (17.3%) |
| 76-100 | 19 (48.7%) | 198 (59.1%) | 25 (46.3%) | 25 (53.2%) | 267 (56.2%) |
| Social functioning |  |  |  |  |  |
| 0-25 | 11 (28.2%) | 94 (28.1%) | 7 (13.0%) | 13 (27.7%) | 125 (26.3%) |
| 26-50 | 7 (17.9%) | 100 (29.9%) | 21 (38.9%) | 17 (36.2%) | 145 (30.5%) |
| 51-75 | 6 (15.4%) | 57 (17.0%) | 10 (18.5%) | 6 (12.8%) | 79 (16.6%) |
| 76-100 | 15 (38.5%) | 84 (25.1%) | 16 (29.6%) | 11 (23.4%) | 126 (26.5%) |
| **Symptom scales** |  |  |  |  |  |
| Fatigue |  |  |  |  |  |
| 0-25 | 8 (20.5%) | 61 (18.2%) | 8 (14.8%) | 6 (12.8%) | 83 (17.5%) |
| 26-50 | 9 (23.1%) | 86 (25.7%) | 11 (20.4%) | 12 (25.5%) | 118 (24.8%) |
| 51-75 | 9 (23.1%) | 80 (23.9%) | 13 (24.1%) | 11 (23.4%) | 113 (23.8%) |
| 76-100 | 13 (33.3%) | 108 (32.2%) | 22 (40.7%) | 18 (38.3%) | 161 (33.9%) |
| Nausea and vomiting |  |  |  |  |  |
| 0-25 | 28 (71.8%) | 255 (76.1%) | 38 (70.4%) | 36 (76.6%) | 357 (75.2%) |
| 26-50 | 8 (20.5%) | 51 (15.2%) | 12 (22.2%) | 9 (19.1%) | 80 (16.8%) |
| 51-75 | 2 (5.1%) | 8 (2.4%) | 1 (1.9%) | 0 (0.0%) | 11 (2.3%) |
| 76-100 | 1 (2.6%) | 21 (6.3%) | 3 (5.6%) | 2 (4.3%) | 27 (5.7%) |
| Pain |  |  |  |  |  |
| 0 | 16 (41.0%) | 146 (43.6%) | 19 (35.2%) | 18 (38.3%) | 199 (41.9%) |
| 33.3 | 9 (23.1%) | 88 (26.3%) | 13 (24.1%) | 12 (25.5%) | 122 (25.7%) |
| 66.6 | 2 (5.1%) | 48 (14.3%) | 10 (18.5%) | 7 (14.9%) | 67 (14.1%) |
| 100 | 12 (30.8%) | 53 (15.8%) | 12 (22.2%) | 10 (21.3%) | 87 (18.3%) |
| Dyspnea |  |  |  |  |  |
| 0 | 19 (48.7%) | 148 (44.2%) | 22 (40.7%) | 22 (46.8%) | 211 (44.4%) |
| 33.3 | 11 (28.2%) | 85 (25.4%) | 11 (20.4%) | 10 (21.3%) | 117 (24.6%) |
| 66.6 | 6 (15.4%) | 62 (18.5%) | 13 (24.1%) | 7 (14.9%) | 88 (18.5%) |
| 100 | 3 (7.7%) | 40 (11.9%) | 8 (14.8%) | 8 (17.0%) | 59 (12.4%) |
| Insomnia |  |  |  |  |  |
| 0 | 17 (43.6%) | 108 (32.2%) | 12 (22.2%) | 20 (42.6%) | 157 (33.1%) |
| 33.3 | 7 (17.9%) | 74 (22.1%) | 14 (25.9%) | 12 (25.5%) | 107 (22.5%) |
| 66.6 | 9 (23.1%) | 77 (23.0%) | 18 (33.3%) | 6 (12.8%) | 110 (23.2%) |
| 100 | 6 (15.4%) | 76 (22.7%) | 10 (18.5%) | 9 (19.1%) | 101 (21.3%) |
| Appetite loss |  |  |  |  |  |
| 0 | 19 (48.7%) | 168 (50.1%) | 23 (42.6%) | 22 (46.8%) | 232 (48.8%) |
| 33.3 | 4 (10.3%) | 56 (16.7%) | 13 (24.1%) | 12 (25.5%) | 85 (17.9%) |
| 66.6 | 8 (20.5%) | 54 (16.1%) | 11 (20.4%) | 3 (6.4%) | 76 (16.0%) |
| 100 | 8 (20.5%) | 57 (17.0%) | 7 (13.0%) | 10 (21.3%) | 82 (17.3%) |
| Constipation |  |  |  |  |  |
| 0 | 24 (61.5%) | 201 (60.0%) | 28 (51.9%) | 29 (61.7%) | 282 (59.4%) |
| 33.3 | 4 (10.3%) | 60 (17.9%) | 9 (16.7%) | 2 (4.3%) | 75 (15.8%) |
| 66.6 | 9 (23.1%) | 48 (14.3%) | 11 (20.4%) | 8 (17.0%) | 76 (16.0%) |
| 100 | 2 (5.1%) | 26 (7.8%) | 6 (11.1%) | 8 (17.0%) | 42 (8.8%) |
| Diarrhea |  |  |  |  |  |
| 0 | 23 (59.0%) | 248 (74.0%) | 38 (70.4%) | 36 (76.6%) | 345 (72.6%) |
| 33.3 | 9 (23.1%) | 48 (14.3%) | 6 (11.1%) | 4 (8.5%) | 67 (14.1%) |
| 66.6 | 6 (15.4%) | 23 (6.9%) | 9 (16.7%) | 6 (12.8%) | 44 (9.3%) |
| 100 | 1 (2.6%) | 16 (4.8%) | 1 (1.9%) | 1 (2.1%) | 19 (4.0%) |
| Financial difficulties* |  |  |  |  |  |
| 0 | 15 (38.5%) | 203 (60.6%) | 23 (42.6%) | 30 (63.8%) | 271 (57.1%) |
| 33.3 | 13 (33.3%) | 60 (17.9%) | 10 (18.5%) | 7 (14.9%) | 90 (18.9%) |
| 66.6 | 8 (20.5%) | 47 (14.0%) | 12 (22.2%) | 4 (8.5%) | 71 (14.9%) |
| 100 | 3 (7.7%) | 25 (7.5%) | 9 (16.7%) | 6 (12.8%) | 43 (9.1%) |

***** Chi-squared-tests for patients with different marital status: p<0.05

^$^ POS and EORTC QLQ-C30 in four equal proportions: Global health status/QoL, functional scales and fatigue; other symptom scales of EORTC QLQ-C30: only four values: 0, 33.3, 66.6, 100; HADS according to clinical classification

POS: Palliative Outcome Scale (range: 0-40, higher values = higher burden)

HADS: Hospital Anxiety and Depression Scale (range: 0-21; higher values = higher burden)

EORTC QLQ C30: European Organization for Research and Treatment of Cancer Quality of Life Questionnaire (higher values = better status for Global health status/QoL and functional scales; higher values = higher burden for symptom scales / items)

**POS free text by marital status, and total**

| **Most relevant problem in the past three days** | **Divorced**  **N=41** | **Single**  **N=29** | **Married**  **N=240** | **Widowed**  **N=34** | **Total**  **N=344** |
| --- | --- | --- | --- | --- | --- |
| Physical symptoms (pain, dyspnea etc.) | 7 (17.1%) | 9 (31.0%) | 65 (27.1%) | 10 (29.4%) | 91 (26.5%) |
| Fear from adverse events (e.g. chemotherapy, radiation) | 3 (7.3%) | 4 (13.8%) | 17 (7.1%) | 8 (23.5%) | 32 (9.3%) |
| Disease and death, dealing with illness | 3 (7.3%) | 1 (3.5%) | 24 (10.0%) | 2 (5.9%) | 30 (8.7%) |
| Life expectancy, prognosis, chances for cure / relief | 5 (12.2%) | 0 (0.0%) | 21 (8.8%) | 2 (5.9 %) | 28 (8.1%) |
| Financial concerns | 3 (7.3%) | 2 (6.9%) | 17 (7.1%) | 1 (2.9%) | 23 (6.7%) |
| No problems | 5 (12.2%) | 2 (6.9%) | 13 (5.4%) | 1 (2.9%) | 21 (6.1%) |
| Miscellaneous problems | 4 (9.8%) | 1 (3.5%) | 13 (5.4%) | 2 (5.9%) | 20 (5.8%) |
| Therapeutic decision | 1 (2.4%) | 0 (0.0%) | 14 (5.8%) | 4 (11.8%) | 19 (5.5%) |
| Concerns about family and relatives | 1 (2.4%) | 0 (0.0%) | 13 (5.4%) | 3 (8.8%) | 17 (4.9%) |
| Home care, autonomy | 4 (9.8%) | 2 (6.9%) | 8 (3.3%) | 1 (2.9%) | 15 (4.4%) |
| Uncertainty and concerns about future | 1 (2.4%) | 2 (6.9%) | 11 (4.6%) | 0 (0.0%) | 14 (4.1%) |
| Psychological burden (depression, anxiety) | 2 (4.9%) | 2 (6.9%) | 7 (2.9%) | 0 (0.0%) | 11 (3.2%) |
| Organization (e.g. transport to hospital, scheduling) | 0 (0.0%) | 2 (6.9%) | 9 (3.8%) | 0 (0.0%) | 11 (3.2%) |
| Concern about work | 2 (4.9%) | 2 (6.9%) | 5 (2.1%) | 0 (0.0%) | 9 (2.6%) |
| Reasons of disease | 0 (0.0%) | 0 (0.0%) | 3 (1.3%) | 0 (0.0%) | 3 (0.9%) |

Problems listed in descending order according to the total number and percent

Percentages refer to number in column
